# Alkynes Hydration in Three‐Component Double‐Acidic Deep Eutectic Solvents

**DOI:** 10.1002/cssc.202501421

**Published:** 2025-11-03

**Authors:** Alessandra Gritti, Valentina Pirovano, Alessandro Caselli, Alejandro Torregrosa‐Chinillach, Matteo Tiecco, Giorgio Abbiati

**Affiliations:** ^1^ Department of Pharmaceutical Sciences General and Organic Chemistry Section “A. Marchesini” University of Milan via Golgi 19 20133 Milano Italy; ^2^ Department of Chemistry University of Milan via Golgi 19 20133 Milano Italy; ^3^ Department of Organic Chemistry and Organic Synthesis Institute (ISO) Alicante University Apdo. 99 03080 Alicante Spain; ^4^ School of Pharma University of Camerino, ChIP Research Center via Madonna delle Carceri 62032 Camerino MC Italy

**Keywords:** alkynes, deep eutectic solvents, hydration, ketones, microwaves

## Abstract

In this work, a sustainable method for the hydration of both terminal and internal alkynes is presented, affording the corresponding ketones with complete Markovnikov regioselectivity. The key advantage of this approach lies in the use of recently developed three‐component, double‐acidic deep eutectic solvents (DESs), designed by our research team, which act as triple‐active media—simultaneously serving as solvents, reagents, and catalysts. The reaction setup is straightforward and can be carried out under conventional or dielectric heating, typically providing the desired products in good to excellent yields. For terminal alkynes, microwave heating further enhances the sustainability of the process by a significant reduction of reaction times. The reusability of the reaction medium is demonstrated through recycling experiments, while the calculation of two green metrics (sE‐factor and EcoScale) highlights the environmental benefits of this strategy.

## Graphical abstract

All‐in‐one solution! Novel three‐component double‐acidic deep eutectic solvents (DESs) enable the regioselective hydration of both terminal and internal alkynes to afford the corresponding ketones. The key factor is the triple activity of the DES: solvent, reagent, and catalyst.

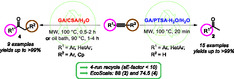

## 1 Introduction

The industrial use of traditional organic solvents has a significant impact on soil, water, and air pollution. In recent decades, considerable efforts have been made to develop organic transformations that occur without the use of solvents (solvent‐free reactions).^[^ ^1^, ^2^, ^3^, ^4^ ^]^ Nevertheless, in many cases, the use of an appropriate medium is crucial to ensure high yields and selectivities. In a chemical reaction, the solvent plays multiple fundamental roles: it dissolves the solute and creates single molecular entities; it enhances the electronic properties of the molecules; and it transfers energy to the atoms, thereby promoting effective molecular collisions with the appropriate energy. An additional function that a medium can display is to directly promote a reaction by acting either as a catalyst or as a reagent. Based on these considerations, it is not surprising that the search for alternative and sustainable solvents for chemical transformations has become an increasingly important topic within the chemical community, both in academic research and industrial applications.^[^ ^5^, ^6^ ^]^

In this context, deep eutectic solvents^[^ ^7^ ^]^ (DESs) represent a modern class of liquids with peculiar physicochemical properties that make them suitable media for organic synthesis.^[^ ^8^, ^9^, ^10^ ^]^ They are characterized by significant features that increase their sustainability, such as low vapor pressures and melting points, low flammability, good potential for reuse and recycling, low cost, ease of preparation, high biodegradability, and low toxicity.^[^ ^11^, ^12^, ^13^ ^]^ The most common type of DESs can be easily prepared by mixing two entities: one acting as a hydrogen‐bond donor, and the other as a hydrogen‐bond acceptor. The interaction between these two entities is responsible for the reduction of the melting point of the resulting deep eutectic mixture, thanks to the impossibility of a regular crystal lattice formation.^[^ ^7^ ^]^ Returning to the role of the solvent in a chemical transformation, DESs are typical examples of media that can act not only as simple, innocent polar solvents but also as reagents or catalysts.

**Scheme 1:**
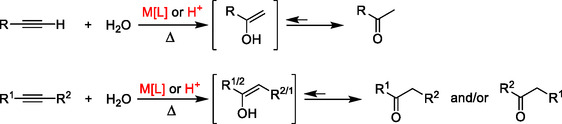
Hydration of terminal and internal alkynes.

A useful and well‐documented approach for the synthesis of carbonyl compounds is the hydration of alkynes (**Scheme** **1**).^[^ ^14^, ^15^, ^16^ ^]^ This reaction involves the formation of an enol intermediate, which rapidly tautomerizes into the more stable carbonyl form. The classic hydration methodology follows Markovnikov's rule, thus terminal alkynes yield the corresponding methyl ketones, whereas the regioselectivity in the case of internal alkynes is more difficult to control and strongly depends on the nature of substituents at the alkyne termini.^[^ ^17^ ^]^

This reaction was first described in 1881 by Kucherov.^[^ ^18^ ^]^ The original protocol required stoichiometric amounts of mercury(II) salts, which are highly undesirable due to their well‐known toxicity.^[^ ^19^ ^]^ A lot of improvements have been made in 150 years of research in the field of synthetic organic chemistry to overcome the limitations of the original approach and avoid the use of poisonous mercury salts. Firstly, the amount of the metal additive has been reduced from stoichiometric to catalytic, and more sustainable metals were introduced. Nowadays, the more advanced hydration protocols involve the use of catalytic amounts of noble metals (Ru, Rh, Pd, Ir, Pt, and Au),^[^ ^20^, ^21^, ^22^ ^]^ whereas only a few approaches use first‐row transition metals.^[^ ^23^ ^]^ At the same time, some metal‐free procedures exploiting the catalytic activity of Brønsted acids such as TfOH or Tf_2_NH,^[^ ^24^ ^]^ PTSA,^[^ ^25^, ^26^, ^27^ ^]^ or concentrated H_2_SO_4_,^[^ ^28^ ^]^ or HCO_2_H^[^ ^29^ ^]^ have been proposed. Notably, many of these procedures exhibit some drawbacks: i) require harsh reaction conditions; ii) display a narrow scope; iii) necessitate robust substrates; and iv) are limited to terminal alkynes. In 2023, a very interesting strategy for the regioselective hydration of terminal alkynes by using a Fe^III^‐based eutectic mixture [FeCl_3_·6H_2_O/Gly (3:1)] as an active medium has been reported by Presa Soto, del Amo, and García‐Álvarez.^[^ ^30^ ^]^ Moreover, the same research group described a modular, three‐component approach to γ‐keto sulfones and γ‐keto phosphine oxides in acidic DES (ChCl/*p*‐TSA·H_2_O) starting from terminal alkynes, aldehydes, and sodium sulfinates or secondary phosphine oxides.^[^ ^31^ ^]^ In this strategy, the hydration of terminal alkynes to methyl ketones represents the first step of a three‐step cascade process.

For many years, our research group has been engaged in the development of alternative and more sustainable strategies for the synthesis of heterocycles and organic scaffolds. Recently, we focused our efforts in this direction by employing more eco‐friendly solvents and reagents. In particular, in some recent works, we reported original DES‐mediated and MW‐enhanced approaches for the synthesis of isocoumarins,^[^ ^32^ ^]^ propargylamines,^[^ ^33^ ^]^ and biindoles.^[^ ^34^ ^]^ The key strategy in these studies was the replacement of the LA/BA catalysts traditionally required to promote the reactions with a carefully selected acidic DES, capable of playing the double role of solvent and catalyst.

**Figure 1:**
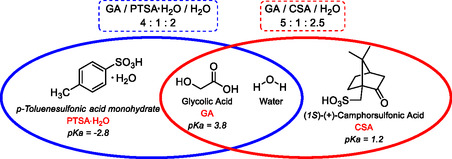
Our three‐component double Brønsted‐acidic DESs.

In our recent work, we reported the synthesis and detailed characterization of a new class of ternary double Brønsted‐acidic DESs, in which water plays the role of the third component (**Figure** **1**).^[^ ^35^ ^]^

The suitability of these DESs as active media in acid‐promoted and metal‐catalyzed organic transformations was briefly investigated in two different reactions chosen as a proof‐of‐concept, i.e., the Claisen‐Schmidt variant of aldol reaction and the gold‐catalyzed synthesis of indoles starting from 2‐alkynylanilines (Arcadi indole synthesis). In both reactions, the DESs were able to catalyze the transformations without the need for additional catalysts.^[^ ^35^ ^]^ Notably, during the study on indole synthesis, we observed the formation of the hydration product of *N*‐protected 2‐(phenylethynyl)aniline as a minor by‐product. Prompted by this serendipitous finding, we decided to explore in depth the potential of our double‐acidic DESs as threefold active media (solvent/catalyst/reagent) in the hydration of terminal and internal alkynes. In this article, we describe the results of our studies.

## 2 Results and Discussion

We began the study by testing the activity of our two double‐acidic DESs on three different terminal arylalkynes (**1a‐c**), each bearing different substituents on the phenyl ring, to evaluate the effect of the presence of electron‐neutral, electron‐donating, and electron‐withdrawing groups on the reaction outcome. The results are reported in **Table** **1**.

**Table 1:** Screening of best reaction condition for hydration of terminal alkynes in double‐acidic DESs.

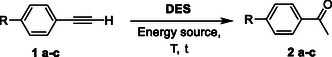							
Entrya)	1‐2	R	DES	t [min]	Energy source	T [°C]	2 Yield %b)
1	**a**	H	GA/PTSA·H_2_O/H_2_O	75c)	Oil bath	90	>99
2	**a**	H	GA/CSA/H_2_O	75c)	Oil bath	90	>99
3	**b**	MeO	GA/PTSA·H_2_O/H_2_O	30c)	Oil bath	90	>99
4	**b**	MeO	GA/CSA/H_2_O	30c)	Oil bath	90	>99
5	**c**	F	GA/PTSA·H_2_O/H_2_O	60c)	Oil bath	90	38
6	**c**	F	GA/CSA/H_2_O	60c)	Oil bath	90	25
7	**a**	H	GA/PTSA·H_2_O/H_2_O	30	MW	100	>99
8	**a**	H	GA/PTSA·H_2_O/H_2_O	10	MW	100	94
9	**a**	H	GA/PTSA·H_2_O/H_2_O	20	MW	100	>99
10	**b**	MeO	GA/PTSA·H_2_O/H_2_O	20	MW	100	95
11	**c**	F	GA/PTSA·H_2_O/H_2_O	20	MW	100	99

a) reaction conditions: sealed vials, 0.4 mmol (**1 a‐c**), 0.5 mL DES. DES type, reaction time, energy source and reaction temperature as reported in the table;

b) yields referred to pure isolated products;

c) reactions stopped after complete consumption of starting material, detected by TLC analysis.

Both double‐acidic DES proved to be highly effective for the hydration of terminal alkynes (Table 1, entries 1–6). The reaction proceeded under relatively mild conditions with complete Markovnikov regioselectivity. The presence of an electron‐neutral or electron‐donating group on the aryl moiety of terminal alkynes is well‐tolerated (e.g., **1a‐b**), and the reaction yields are quantitative (Table 1, entries 1–4). Conversely, the presence of a strong inductive electron‐withdrawing group led to lower yields, although the regioselectivity remained complete. Interestingly, the GA/PTSA/H_2_O‐based DES appeared to provide better results with terminal alkynes (Table 1, compare entries 5 and 6). To improve the reaction yields for electron‐poor terminal alkyne **1c** and reduce reaction times, we switched to a more efficient energy source such as dielectric heating (Table 1, entries 7–11). We investigated the minimum time to obtain a quantitative conversion of alkyne **1a** (Table 1, entries 7–9), and we identified 20 mins as optimal reaction time under microwave heating. These conditions proved to be optimal also for electron‐reach (**1b**) and electron‐poor (**1c**) terminal alkynes (Table 1, entries 10 and 11). Notably, the role of the reaction medium in these transformations is threefold: solvent, catalyst (acidic network), and reagent (water component).

**Scheme 2:**
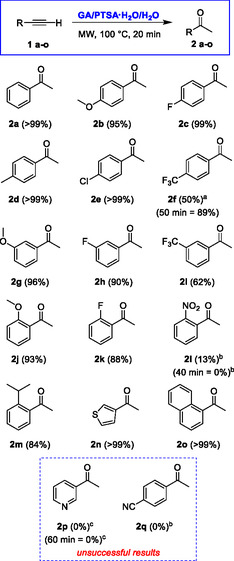
Scope and limitations of MW‐enhanced hydration of terminal alkynes in GA/PTSA·H_2_O/H_2_O‐based DES (isolated yields; ^a)^40% unreacted starting material; ^b)^beside a mixture of unidentified by‐products; ^c)^starting material quantitatively recovered).

With the optimized methodology in hand, we further explored the scope and limitations of the approach by changing not only the electronic nature of the substituent on the phenyl ring but also its position (**Scheme** **2**).

The approach allows a wide set of different substituents on the phenyl group. Neutral groups and EDGs in any position of the ring are well tolerated (**2d, 2e, 2g**, **2j**, and **2m**), as are proximate annulation (**2o**), and electron‐rich heterocyclic systems (**2n**). Regardless of their position, strong electron‐withdrawing groups appear to only weakly affect the reaction outcome (**2h**, **2i** and **2k**). In some cases, a prolonged reaction time allows for increase in a modest reaction yield (i.e.,  **2f**). Some electron‐withdrawing substituents, such as nitro and cyano groups, despite their position, seem to be critical, and the reaction of the corresponding alkyne gave a very low yield (**2l**) or failed (**2q**), generating a complex mixture of unidentified by‐products. Finally, the hydration of an alkyne characterized by the presence of a strong electron‐poor heterocycle such as pyridine failed even after a prolonged reaction time, and the unreacted starting material was quantitatively recovered (**2p**). All methylketones **2a–o**, obtained from terminal alkynes, were isolated in sufficient purity through simple liquid–liquid extraction, with no need for further chromatographic purification, thereby enhancing the overall sustainability of the approach.

As stated in the introduction, two major limitations of existing methods for the hydration of alkynes are their poor efficiency with internal alkynes and the associated challenges in achieving regioselectivity. Therefore, in the next part of this study, we faced the hydration on internal alkynes using 1‐methoxy‐4‐(*p*‐tolylethynyl)benzene **3a** and 1‐fluoro‐4‐(*p*‐tolylethynyl)benzene **3b** as model compounds. The results of the screening for the best reaction conditions are shown in **Table** **2**.

**Table 2:** Screening of best reaction condition for hydration of internal alkynes in double‐acidic DESs.

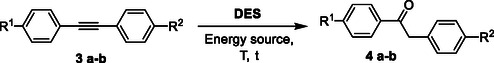								
Entrya)	3‐4	R^1^	R^2^	DES	t [min]	Energy source	T [°C]	4 Yield %b)
1	**a**	MeO	Me	GA/PTSA·H_2_O/H_2_O	60c)	Oil bath	90	77
2	**a**	MeO	Me	GA/CSA/H_2_O	60c)	Oil bath	90	85
3	**b**	Me	F	GA/PTSA·H_2_O/H_2_O	120c)	Oil bath	90	49
4	**b**	Me	F	GA/CSA/H_2_O	225c)	Oil bath	90	66
5	**a**	MeO	Me	GA/CSA/H_2_O	30	MW	100	82
6	**b**	Me	F	GA/CSA/H_2_O	60	MW	100	95

a) reaction conditions: sealed vials, 0.2 mmol (**3 a‐b**), 0.5 mL DES. DES type, reaction time, energy source and reaction temperature as reported in the table;

b) yields referred to pure isolated products;

c) reactions stopped after complete consumption of starting material detected by TLC analysis.

**Scheme 3:**
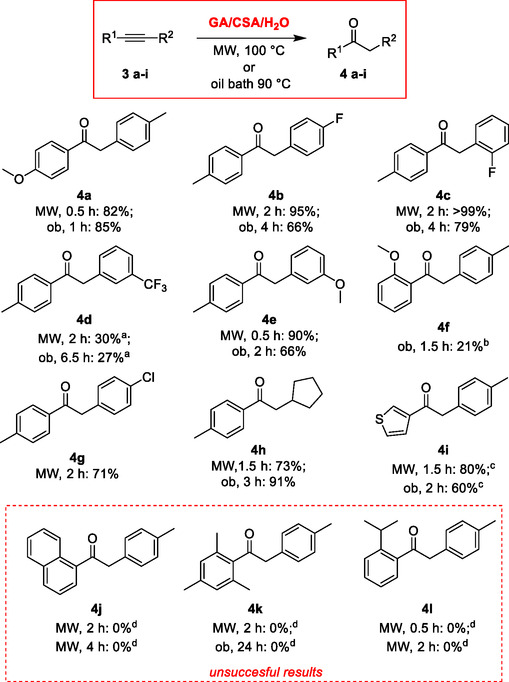
Scope and limitations of MW‐enhanced hydration of internal alkynes in GA/CSA/H_2_O‐based DES (isolated yields; ^a)^36% unreacted starting material; ^b)^beside 62% of the corresponding 2‐*p*‐tolylbenzofuran; ^c)^10% of the regioisomeric 2‐(thiophen‐3‐yl)‐1‐(*p*‐tolyl)ethan‐1‐one; ^d)^starting material quantitatively recovered).

At 90 °C under traditional heating, both double‐acidic DESs were also able to transform also internal alkynes **3a** and **3b** into the corresponding ketones **4a** and **4b** in a regiospecific way (Table 2, entries 1–4). Not surprisingly, the reactions of electron‐rich alkynes proceeded faster and gave higher yields. Interestingly, for internal alkynes, the GA/CSA/H_2_O‐based DES appeared to be the medium of choice (Table 2, compare entries 1–2 and 3–4). A slight increase in the reaction temperature under dielectric heating allowed to reduce the reaction time for the electron‐rich alkyne (Table 2 entry 5) and improve the yields for electron‐poor ones (Table 2 entry 6). In these cases as well, the approach showed complete Markovnikov selectivity. **Scheme** **3** illustrates the scope and limitations of the optimized protocol for internal alkynes.

The hydration of internal alkynes gave good results, with more variable yields ranging from modest to very good. These outcomes were strongly influenced by reaction time and the energy source used. In general, dielectric heating provided better or comparable results in shorter reaction times. However, the advantages of microwave irradiation appeared less pronounced for internal alkynes than for terminal ones, possibly due to the variability in optimal reaction times. The behavior of internal alkynes proved to be less predictable and standardizable, showing greater sensitivity to the nature of the substituents. Despite this variability, the reactions of internal alkynes proved to be highly regioselective, with selectivity mainly governed by the electronic factors related to the type and the position of the substituents on the aryl ring at the alkyne termini. No straightforward correlation emerged between the electronic nature of the substituents and the reaction yields. The reaction is also effective for alkyl–aryl alkynes, affording the corresponding 1‐aryl‐2‐alkylethanone **4h** regioselectively and in good yield. Nevertheless, bulky electron‐donating ortho substituents on phenyl ring strongly hamper the reactivity, as demonstrated by the unsuccessful results **4j‐l**. On the other hand, the difference in the regioselectivity observed in the reaction of the alkynes bearing a *para*/*ortho* (**4a** and **4f**) versus *meta* (**4e**) methoxyphenyl substituents is a nice example of the well‐known prevalence of mesomeric (+M) over inductive (‐I) effects. In contrast to methylketones **2a–o**, the purification of 1,2‐substituted ethanones **4a–i** required flash‐column chromatography.

It is worth underlining that, compared to previously reported and closely related acid‐promoted approaches, the use of our ternary double‐acidic deep eutectic mixture offers notable advantages. For instance, the TfOH/Tf_2_NH‐catalyzed method described by Shirakawa and coworkers^[^ ^24^ ^]^ in 2000 requires a hazardous solvent such as 1,4‐dioxane,^[^ ^36^ ^]^ prolonged reaction times (up to 78 h), and generally affords lower yields (e.g., **2b**: 95% vs. 86%; **2d**: >99% vs. 85%; **2e**: >99% vs. 63%; **2f**: 89% vs. 48%). Similarly, the group of Alami, in three different reports, described the hydration of unsymmetrical arylalkynes using PTSA monohydrate in EtOH,^[^ ^27^ ^]^ also under dielectric heating.^[^ ^25^, ^26^ ^]^ In this case as well, the reactions required either prolonged heating under conventional conditions (up to 144 h) or higher temperatures (up to 170 °C) under MW irradiation, especially for the scarcely studied diarylalkynes. Moreover, some regioselectivity issues were observed, and the method proved completely unsuitable for terminal alkynes.

To gain more insight into the possible synergistic effect of the double‐acidic components of the eutectic solvent, we carried out a series of control experiments (**Table** **3**).

**Table 3:** Control experiments.

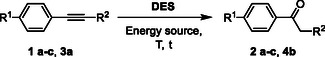								
Entrya)	–	R^1^	R^2^	DESb)	t [min]	Energy source	T [°C]	2/4 (Yield [%])c)
1	**1a**	H	H	ChCl/PTSA·H_2_O	20	MW	100	**2a** (78)
2	**1b**	MeO	H	ChCl/PTSA·H_2_O	20	MW	100	**2b** (98)
3	**1c**	F	H	ChCl/PTSA·H_2_O	20	MW	100	**2c** (95)
4	**3a**	MeO	4‐Me‐Ph	CSA/CyMes (1:1)	60	Oil bath	90	**4a** (n.r.)d)
5	**3a**	MeO	4‐Me‐Ph	CSA/CyMes/H_2_O (1:1:1.5)	60	Oil bath	90	**4a** (traces)d)
6	**3a**	MeO	4‐Me‐Ph	PTSA·H_2_O/BTMAMes (1:1)	120	Oil bath	90	**4a** (30)e)

a) reaction conditions: sealed vials, 0.4 mmol (**1 a‐c**) or 0.2 mmol (**3a**), 0.5 mL DES. DES type, reaction time, energy source and reaction temperature as reported in the table;

b) ChCl = choline chloride, CyMes = cyclohexyltrimethylammonium mesylate, BTMAMes = benzyltrimethylammonium mesylate;

c) yields referred to pure isolated products;

d) unreacted starting material almost quantitatively recovered;

e) beside a mixture of unidentified by‐product and unreacted starting material.

First, the hydration of three differently substituted terminal alkynes **1a–c** was performed under the optimized reaction conditions but using the well‐known choline chloride‐based single‐acidic eutectic mixture ChCl/PTSA·H_2_O. In all cases, the reaction afforded the desired products in high yields, comparable or only slightly lower than those obtained using the double‐acidic DES (cf. Table 1, entries 9–11 vs. Table 3, entries 1–3). In contrast, the reactions of internal alkyne **3a** in representative single‐acidic DES based on CSA or PTSA either failed (Table 3, entries 4 and 5) or gave the corresponding ketones in low yield (Table 3, entry 6), even after the addition of 1.5 equivalents of water (Table 3, entry 5). These results highlight the crucial and cooperative role of the two acidic components of the eutectic mixture for the successful hydration of alkynes—particularly internal alkynes—a result that cannot be replicated by DESs containing only a single‐acidic component, even in the presence of added water.

**Figure 2:**
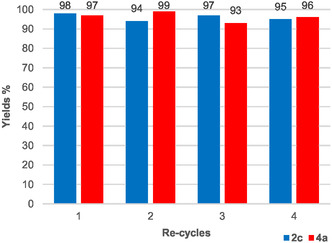
Recycling studies on the synthesis of 1‐(4‐fluorophenyl)ethan‐1‐one (**2c**) and 1‐(4‐methoxyphenyl)‐2‐(*p*‐tolyl)ethan‐1‐one (**4a**).

The reusability of the medium and the scalability of the process represent important advances toward enhancing the sustainability of new synthetic chemical methodologies. To investigate this aspect, the reactions of terminal alkyne **1c** and internal alkyne **3a** were scaled up fivefold to 2 and 1 mmol, respectively, in 5 mL of the suitable DES. After each cycle, the DES was poured into deionized water, and the product was extracted twice with diethyl ether (for **2c**) or ethyl acetate (for **4a**). When possible, ethyl acetate was chosen for its safety and low impact on the environment.^[^ ^37^, ^38^ ^]^ The excess of water evaporated under reduced pressure to restore the initial volume of the DES. Variations in the water content of the DES are not critical, as it has been demonstrated that the eutectic nature of the mixture is maintained over a relatively wide range of water concentrations.^[^ ^35^ ^]^ Following this procedure, the eutectic mixtures could be reused for up to four consecutive runs without a significant loss in yield (**Figure** **2**).

The green profile of a chemical transformation can be estimated through the calculation of green metrics. Numerous metrics are available, but not all are suitable for early laboratory‐stage studies, and some have yet to reach broad acceptance.^[^ ^39^ ^]^ To evaluate the greenness of our approach, we selected Sheldon's environmental factor^[^ ^40^, ^41^ ^]^ and the EcoScale^[^ ^42^ ^]^ (see Supporting Information for details). In particular, we decided to calculate the simple E‐factor $\left(\text{sEF}=\frac{\sum \text{Raw}\;\text{materials}+\sum \text{reagents }-\text{Product}}{\text{Product}}\right)$ which is ideal for early‐phase process route scouting.^[^ ^39^, ^43^ ^]^ The EcoScale, on the other hand, provides a comprehensive evaluation of sustainability, starting from an ideal score of 100 points and subtracting penalty points based on reaction yield, cost, and safety of reagents, and complexity of setup, workup, and purification.

The calculated sE‐factors for the single‐run synthesis of acetophenone **2a** and 1‐(4‐methoxyphenyl)‐2‐(*p‐*tolyl)ethan‐1‐one **4a** are 13.81 and 16.66, respectively. These values are in line with those typically reported for the preparation of fine chemicals.^[^ ^40^, ^41^ ^]^ Moreover, the sE‐factor for the syntheses of **2c** and **4a** under recycling conditions (4 runs) became particularly attractive, 7.36 and 9.89, respectively.

The EcoScale for the single‐run synthesis of acetophenone **2a** and 1‐(4‐methoxyphenyl)‐2‐(*p*‐tolyl)ethan‐1‐one **4a** yielded valuable scores of 88 and 74.5, respectively, where values greater than 75 are considered excellent.^[^ ^42^ ^]^

## 3 Conclusion

In summary, we have developed a novel and sustainable strategy for the regiospecific hydration of both terminal and internal alkynes, affording the corresponding ketones with high efficiency. The key innovation of this methodology lies in the use of original three‐component acidic aqueous DESs, which uniquely serve a triple function—as reaction media, catalysts, and reagents. This multifaceted role significantly contributes to the atom economy and operational simplicity of the process. The reaction setup is straightforward, operates under mild conditions without the need for inert atmosphere or stringent conditions, and is compatible with both conventional and dielectric heating. Notably, the transformation of terminal alkynes does not need chromatographic purification. Overall, the method delivers good to excellent yields, reaching quantitative conversions in several cases. The active DES systems demonstrate excellent reusability over multiple cycles without appreciable loss in catalytic performance. Importantly, the sustainability of the protocol is supported by favorable values in two different green metrics: a low sE‐factor (in particular, when the DES is recycled) and a high EcoScale score, underscoring the environmental and practical advantages of this approach.

## 4 Experimental Section

4.1

4.1.1

##### General Procedure for the Synthesis of Methylketones 2a‐o

In a MW vial, the appropriate alkyne **1a‐o** was added to the selected DES (GA/pTSA·H_2_O/H_2_O, 0.5 mL), and the mixture was heated at 100 °C under MW irradiation for the appropriate time. Upon completion, the reaction mixture was diluted with H_2_O (20 mL) and extracted with Et_2_O (3 × 10 mL). The combined organic layers were washed with saturated NaHCO_3_ solution (20 mL) and brine (20 mL), dried over Na_2_SO_4_, filtered, and concentrated under reduced pressure to afford the corresponding ketone.

##### General Procedures for the Synthesis of Internal Ketones 4a‐i. Method a

In a screw capped vial, the appropriate alkyne **3a‐i** was added to the selected DES (GA/CSA/H_2_O, 0.5 mL), and the mixture was heated at 90 °C in an oil bath. Upon completion, as monitored by TLC, the reaction mixture was diluted with H_2_O (20 mL) and extracted with Et_2_O (3 × 10 mL). The combined organic layers were washed with saturated NaHCO_3_ solution (20 mL) and brine (20 mL), dried over Na_2_SO_4_, filtered, and concentrated under reduced pressure. The crude reaction mixture was purified by flash‐column chromatography on silica gel to afford the corresponding ketone. Method B: In a MW vial, the appropriate alkyne 3**a‐i** was added to the selected DES (GA/CSA/H_2_O, 0.5 mL), and the mixture was heated at 100 °C under MW irradiation for the appropriate time. Upon completion, as monitored by TLC, the reaction mixture was diluted with H_2_O (20 mL) and extracted with EtOAc (3 × 10 mL). The combined organic layers were washed with saturated NaHCO_3_ solution (20 mL) and brine (20 mL), dried over Na_2_SO_4_, filtered, and concentrated under reduced pressure. The crude reaction mixture was purified by flash‐column chromatography on silica gel to afford the corresponding ketone.

##### Recycle Trial for the Synthesis of 2c

In a MW vial, the alkyne **1c** (2 mmol, 240 mg) was added to the selected DES (GA/pTSA·H_2_O/H_2_O, 5 mL), and the mixture was heated at 100 °C under MW irradiation for 20 min. Upon completion, the reaction mixture was diluted with deionized H_2_O (20 mL) and extracted with Et_2_O (2 × 10 mL). The combined organic layers were washed with saturated NaHCO_3_ solution (20 mL) and brine (20 mL), dried over Na_2_SO_4_, filtered, and concentrated under reduced pressure to afford the corresponding ketone **2c**. The aqueous phase was evaporated under reduced pressure to restore the initial volume of the DES. Variations in the water content of the DES are not critical, as it has been demonstrated that the eutectic nature of the mixture is maintained over a relatively wide range of water concentrations.^[^ ^3^ ^]^ The DES was then used for a new cycle.

##### Recycle Trial for the Synthesis of 1‐(4‐Methoxyphenyl)‐2‐(p‐Tolyl)ethan‐1‐one 4a

In a screw capped vial, the alkyne **2a** (1 mmol, 222 mg) was added to the selected DES (GA/CSA/H_2_O, 5 mL), and the mixture was heated at 90 °C in an oil bath. Upon completion, as monitored by TLC, the reaction mixture was diluted with deionized H_2_O (20 mL) and extracted with EtOAc (2 × 10 mL). The combined organic layers were washed with saturated NaHCO_3_ solution (20 mL) and brine (20 mL), dried over Na_2_SO_4_, filtered, and concentrated under reduced pressure to afford the corresponding ketone **4a**. The aqueous phase was evaporated under reduced pressure to restore the initial volume of the DES. Variations in the water content of the DES are not critical, as it has been demonstrated that the eutectic nature of the mixture is maintained over a relatively wide range of water concentrations.^[^ ^35^ ^]^ The DES was then used for a new cycle.

## Conflict of Interest

The authors declare no conflict of interest.

## Supporting information

Supplementary Material

## Data Availability

The data that support the findings of this study are available in the supplementary material of this article.
