# Supplementary material for: Alkynes Hydration in Three‐Component Double‐Acidic Deep Eutectic Solvents
Source: ChemSusChem. 2025 Nov 3;19(1):e202501421. doi: 10.1002/cssc.202501421 (PMC12767271; doi:10.1002/cssc.202501421)
Supplement: Supplementary file 1 — Supplementary Material [file CSSC-19-e202501421-s001.pdf]

Supporting Information

Alkynes Hydration in Three-Component Double-Acidic Deep Eutectic Solvents

Alessandra Gritti,[a,b] Valentina Pirovano,[a] Alessandro Caselli,[b] Alejandro Torregrosa-Chinillach,[c] Matteo Tiecco,[d] Giorgio Abbiati[a]*

[a] *Department of Pharmaceutical Sciences, General and Organic Chemistry Section "A. Marchesini", University of Milan, via Golgi 19, 20133 Milano, Italy.*
[b] *Department of Chemistry, University of Milan, via Golgi 19, 20133 Milano, Italy.*
[c] *Department of Organic Chemistry and Organic Synthesis Institute (ISO), Alicante University, Apdo. 99, 03080 Alicante, Spain.*
[d] *School of Pharma, University of Camerino, ChIP Research Center, via Madonna delle Carceri, 62032 Camerino, MC, Italy.*

Table of contents

General remarks	S-2
General procedure for the synthesis of internal alkynes **3d,e,h,i**	S-2
General procedure for the synthesis of methylketones **2a-o**	S-4
General procedure for the synthesis of internal ketones **4a-i**: Methods A and B	S-6
Recycle trial for the synthesis of **2c**	S-8
Recycle trial for the synthesis of **4a**	S-8
Calculations of Simple E-Factor (sEF)	S-9
Calculations of EcoScale	S-10
References	S-11
^{1}H and ^{13}C spectra of compounds	S-12

General remarks

All chemicals and solvents were purchased from Merck®, BLDPharm®, and Zentek®, and were used without further purification. The chromatographic column separations were performed by flash technique, using silica gel (pore size 60 Å, particle size 230–400 mesh, Merck Grade 9385). For thin-layer chromatography (TLC), Silica on TLC Alu foils with a fluorescent indicator (254 nm) was employed and the detection was performed by irradiation with UV light (λ = 254 nm and/or 366 nm).^{1}H NMR analyses were performed with a 300 MHz (Varian®) or 400 MHz (Bruker®) spectrometer at room temperature. The coupling constants (*J*) are expressed in Hertz (Hz), the chemical shifts (δ) in ppm. The multiplicity of the proton spectra was described by the following abbreviations: s (singlet), d (doublet), t (triplet), q (quartet), p (quintet), dt (double triplet), dd (double doublet), m (multiplet), br (broad). Microwave-enhanced reactions were performed with the single-mode microwave synthesizer "Biotage® Initiator Classic". Compounds **1a-l,n-q** were purchased from standard chemical suppliers and directly used without further purification. Compounds **1m**[1] and **3a-c,f,g,j**[2] were prepared according to literature procedures. Products **2a-o** and **4a,b,e-h** are known compounds and were identified by comparison with spectral data reported in the cited references. The preparation and full characterization of our three-component double Brønsted-acidic DESs have been in detail described in our previous paper.[3]

General procedure for the synthesis of internal alkynes 3d,e,h,i.

Under a nitrogen atmosphere, to a stirred solution of *trans*-dichlorobis-(triphenylphosphine)palladium(II) ($PdCl_2(PPh_3)_2$, 1 mol%) and 4-methyl-1-iodobenzene (1 equiv.) in anhydrous TEA (0.3 M), the appropriate alkyne (1.2 equiv.) was added. The reaction mixture was stirred at rt for 5 min, then CuI (1 mol%) was added. Stirring was continued at rt until TLC analysis indicated complete consumption of the starting material. The reaction mixture was filtered through a thin pad of Celite, and the solvent was removed at reduced pressure. The crude reaction mixture was purified by flash column chromatography on silica gel to afford the desired product **3d,e,h,i.**

1-(*p*-tolylethynyl)-3-(trifluoromethyl)benzene 3d

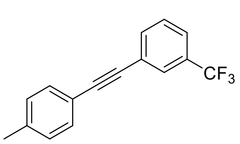

The general procedure was followed using: 1-ethynyl-3-(trifluoromethyl)benzene (470 mg, 2.8 mmol), 4-methyl-1-iodobenzene (500 mg, 2.5 mmol), $PdCl_2(PPh_3)_2$ (16 mg, 0.02 mmol) and CuI (4 mg, 0.02 mmol). Reaction time: 1 h. Purification of the crude by flash column chromatography (SiO_2, Hex/EtOAc 98:2) afforded **3d** (584 mg, 97%) as a white solid. ^{1}H NMR (300 MHz, $CDCl_3$): 7.78 (tt, *J* = 1.6, 0.8 Hz, 1H), 7.71 – 7.65 (m, 1H), 7.59 – 7.54 (m, 1H), 7.50 – 7.40 (m, 3H), 7.21 – 7.12 (m, 2H), 2.38 (s, 3H). Spectral data are in good agreement with literature values.[4]

1-methoxy-3-(*p*-tolylethynyl)benzene 3e

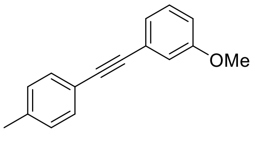

The general procedure was followed using: 1-ethynyl-3-methoxybenzene (370 mg, 2.8 mmol), 4-methyl-1-iodobenzene (500 mg, 2.5 mmol), $PdCl_2(PPh_3)_2$ (16 mg, 0.02 mmol) and CuI (4 mg, 0.02 mmol). Reaction time: 1 h. Purification of the crude by flash column chromatography (SiO_2, Hex/EtOAc 98:2 -> 95:5) afforded **3e** (494 mg, 96%) as a white solid. ^{1}H NMR (300 MHz, $CDCl_3$): 7.43 (d, *J* = 8.1 Hz, 2H), 7.28 – 7.21 (m, 1H), 7.19 – 7.09 (m, 3H), 7.05 (dd, *J* = 2.7, 1.4 Hz, 1H), 6.88 (ddd, *J* = 8.3, 2.7, 1.1 Hz, 1H), 3.83 (s, 3H), 2.37 (s, 3H). Spectral data are in good agreement with literature values.[5]

1-(cyclopentylethynyl)-4-methylbenzene 3h

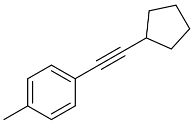

The general procedure was followed using: ethynylcyclopentane (264 mg, 2.8 mmol), 4-methyl-1-iodobenzene (500 mg, 2.5 mmol), $PdCl_2(PPh_3)_2$ (16 mg, 0.02 mmol) and CuI (4 mg, 0.02 mmol). Reaction time: 2 h. Purification of the crude by flash column chromatography (SiO_2, Hex/EtOAc 99:1) afforded **3h** (383 mg, 83%) as a transparent oil. ^{1}H NMR (300 MHz, $CDCl_3$): 7.29 (d, *J* = 7.0 Hz, 2H), 7.08 (d, *J* = 8.0 Hz, 2H), 2.88 – 2.73 (m, 1H), 2.33 (s, 3H), 2.07 – 1.90 (m, 2H), 1.83 – 1.53 (m, 7H). Spectral data are in good agreement with literature values.[6]

2-(*p*-tolylethynyl)thiophene 3i

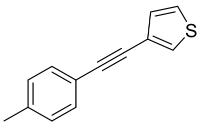

The general procedure was followed using: 3-ethynylthiophene (303 mg, 2.8 mmol), 4-methyl-1-iodobenzene (500 mg, 2.5 mmol), $PdCl_2(PPh_3)_2$ (16 mg, 0.02 mmol) and CuI (4 mg, 0.02 mmol). Reaction time: 1 h. Purification of the crude by flash column chromatography (SiO_2, Hex/EtOAc 98:2) afforded **3i** (475 mg, 100%) as a white solid. ^{1}H NMR (300 MHz, $cdcl_3$) δ 7.50 (dd, *J* = 3.0, 1.2 Hz, 1H), 7.43 – 7.38 (m, 2H), 7.29 (dd, *J* = 5.0, 3.0 Hz, 1H), 7.19 (dd, *J* = 5.0, 1.2 Hz, 1H), 7.17 – 7.11 (m, 2H), 2.36 (s, 3H). Spectral data are in good agreement with literature values.[7]

1,3,5-trimethyl-2-(*p*-tolylethynyl)benzene 3k

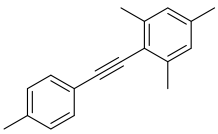

The general procedure was followed using: 2-ethynyl-1,3,5-trimethylbenzene (412 mg, 2.85 mmol), 4-methyl-1-iodobenzene (500 mg, 2.5 mmol), $PdCl_2(PPh_3)_2$ (16 mg, 0.02 mmol) and CuI (4 mg, 0.02 mmol). Reaction time: 12 h. Purification of the crude by flash column chromatography (SiO_2, Hex/EtOAc 99:1) afforded **3k** (597 mg, 98%) as a white solid. ^{1}H NMR (300 MHz, $CDCl_3$): 7.43 (d, *J* = 8.1 Hz, 2H), 7.16 (d, *J* = 7.8 Hz, 2H), 6.89 (s, 2H), 2.47 (s, 6H), 2.37 (s, 3H), 2.29 (s, 3H). Spectral data are in good agreement with literature values. [8]

1-isopropyl-2-(*p*-tolylethynyl)benzene 3l

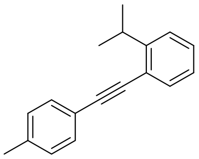

The general procedure was followed using: 1-ethynyl-2-isopropylbenzene (411 mg, 2.85 mmol), 4-methyl-1-iodobenzene (500 mg, 2.5 mmol), $PdCl_2(PPh_3)_2$ (16 mg, 0.02 mmol) and CuI (4 mg, 0.02 mmol). Reaction time: 12 h. Purification of the crude by flash column chromatography (SiO_2, Hex) afforded **3l** (468 mg, 81%) as a white solid. ^{1}H NMR (300 MHz, $CDCl_3$): 7.50 (dd, *J* = 7.4, 1.2 Hz, 1H), 7.43 (d, *J* = 8.0 Hz, 2H), 7.30 (d, *J* = 4.9 Hz, 2H), 7.17 (d, *J* = 8.9 Hz, 3H), 3.55 (pd, *J* = 7.2, 1.4 Hz, 1H), 2.38 (s, 3H), 1.31 (d, *J* = 6.9, 6H). Spectral data are in good agreement with literature values.[9]

General procedure for the synthesis of methylketones 2a-o

In a MW vial, the appropriate alkyne **1a-o** was added to the selected DES (GA/pTSA·H_2O/H_2O, 0.5 mL), and the mixture was heated at 100 °C under MW irradiation for the appropriate time. Upon completion, the reaction mixture was diluted with H_2O (20 mL) and extracted with Et_2O (3 × 10 mL). The combined organic layers were washed with saturated $NaHCO_3$ solution (20 mL) and brine (20 mL), dried over Na_2SO_4, filtered, and concentrated under reduced pressure to afford the corresponding ketone.

Acetophenone 2a

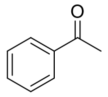

Synthetized following the general procedure from acetylene **1a** (41 mg, 0.4 mmol). Reaction time 20 min. **2a** was obtained in 100% yield (48 mg). ^{1}H NMR (300 MHz, $CDCl_3$): 7.99 – 7.94 (m, 2H), 7.61 – 7.53 (m, 1H), 7.50 – 7.43 (m, 2H), 2.61 (s, 3H). Spectral data are in good agreement with literature values.[10]

1-(4-Methoxyphenyl)ethan-1-one 2b

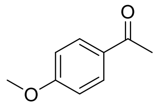

Synthetized following the general procedure from 1-ethynyl-4-methoxybenzene **1b** (53 mg, 0.4 mmol). Reaction time 20 min. **2b** was obtained in a 95% yield (57 mg), as a orange solid. ^{1}H NMR (300 MHz, $CDCl_3$): 7.97 – 7.90 (m, 2H), 6.97 – 6.90 (m, 2H), 3.87 (s, 3H), 2.56 (s, 3H). Spectral data are in good agreement with literature values.[9]

1-(4-Fluorophenyl)ethan-1-one 2c

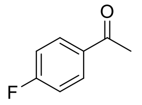

Synthetized following the general procedure from 1-ethynyl-4-fluorobenzene **1c** (48 mg, 0.4 mmol). Reaction time 20 min. **2c** was obtained in 76% yield (76 mg) as a viscous yellow solid. ^{1}H NMR (300 MHz, $CDCl_3$): 8.02 – 7.94 (m, 2H), 7.17 – 7.09 (m, 2H), 2.59 (s, 3H). Spectral data are in good agreement with literature values. [9]

1-(*p*-Tolyl)ethan-1-one 2d

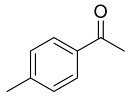

Synthetized following the general procedure from 1-ethynyl-4-methylbenzene **1d** (46 mg, 0.4 mmol). Reaction time 20 min. **2d** was obtained in 100% yield (54 mg) as a yellow liquid. ^{1}H NMR (300 MHz, $CDCl_3$): 7.97 – 7.77 (m, 2H), 7.30 – 7.19 (m, 2H), 2.57 (s, 3H), 2.40 (s, 3H). Spectral data are in good agreement with literature values. [9]

1-(4-Chlorophenyl)ethan-1-one 2e

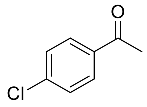

Synthetized following the general procedure from 1-ethynyl-4-chlorobenzene **1e** (55 mg, 0.4 mmol). Reaction time 20 min. **2e** was obtained in 100% yield (62 mg) as a yellow liquid. ^{1}H NMR (300 MHz, $CDCl_3$): 7.98 – 7.81 (m, 2H), 7.51 – 7.40 (m, 2H), 2.60 (s, 3H). Spectral data are in good agreement with literature values. [9]

1-(4-(Trifluoromethyl)phenyl)ethan-1-one 2f

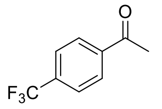

Synthetized following the general procedure from 1-ethynyl-4-(trifluoromethyl)benzene **1f** (68 mg, 0.4 mmol). Reaction time 50 min. **2f** was obtained in 89% yield (67 mg) as a yellow liquid. ^{1}H NMR (300 MHz, $CDCl_3$): 8.12 – 8.02 (m, 2H), 7.74 (d, J = 8.8 Hz, 2H), 2.65 (s, 3H). Spectral data are in good agreement with literature values.[11]

1-(3-Methoxyphenyl)ethan-1-one 2g

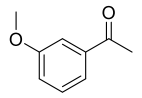

Synthetized following the general procedure from 1-ethynyl-3-methoxybenzene **1g** (53 mg, 0.4 mmol). Reaction time 20 min. **2g** was obtained in 96% yield (58 mg) as a yellow liquid. ^{1}H NMR (300 MHz, $CDCl_3$): 7.56 – 7.46 (m, 2H), 7.40 – 7.32 (m, 1H), 7.10 (ddt, *J* = 8.2, 2.6, 0.8 Hz, 1H), 3.85 (s, 3H), 2.59 (s, 3H). Spectral data are in good agreement with literature values.[10]

1-(3-Fluorophenyl)ethan-1-one 2h

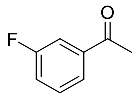

Synthetized following the general procedure from 1-ethynyl-3-fluorobenzene **1h** (48 mg, 0.4 mmol). Reaction time 20 min. **2h** was obtained in 90% yield (50 mg) as a yellow liquid. ^{1}H NMR (300 MHz, $CDCl_3$): 7.76 – 7.70 (m, 1H), 7.67 – 7.59 (m, 1H), 7.50 – 7.38 (m, 1H), 7.31 – 7.21 (m, 1H), 2.60 (d, *J* = 0.6 Hz, 3H). Spectral data are in good agreement with literature values. [12]

1-(3-(Trifluoromethyl)phenyl)ethan-1-one 2i

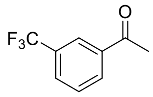

Synthetized following the general procedure from 1-ethynyl-3-(trifluoromethyl)benzene **1i** (68 mg, 0.4 mmol). Reaction time 20 min. **2i** was obtained in 62% yield (47 mg) as a yellow liquid. ^{1}H NMR (300 MHz, $CDCl_3$): 8.20 (s, 1H), 8.13 (d, *J* = 7.8 Hz, 1H), 7.81 (d, *J* = 8.4 Hz, 1H), 7.64 – 7.57 (m, 1H), 2.64 (s, 3H). Spectral data are in good agreement with literature values.[13]

1-(2-Methoxyphenyl)ethan-1-one 2j

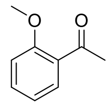

Synthetized following the general procedure from 1-ethynyl-2-methoxybenzene **1j** (53 mg, 0.4 mmol). Reaction time 20 min. **2j** was obtained in 99% yield (60 mg) as a green liquid. ^{1}H NMR (300 MHz, $CDCl_3$): 7.72 (dd, *J* = 7.7, 1.9 Hz, 1H), 7.49 – 7.41 (m, 1H), 7.02 – 6.93 (m, 2H), 3.89 (s, 3H), 2.60 (s, 3H). Spectral data are in good agreement with literature values.[10]

1-(2-Fluorophenyl)ethan-1-one 2k

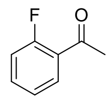

Synthetized following the general procedure from 1-ethynyl-2-fluorobenzene **1k** (48 mg, 0.4 mmol). Reaction time 20 min. **2k** was obtained in 88% yield (48 mg) as a yellow oil. ^{1}H NMR (300 MHz, $CDCl_3$): 7.91 – 7.80 (m, 1H), 7.55 – 7.43 (m, 1H), 7.23 – 7.04 (m, 2H), 2.63 (d, *J* = 4.9 Hz, 3H). Spectral data are in good agreement with literature values.[14]

1-(2-Nitrophenyl)ethan-1-one 2l

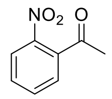

Synthetized following the general procedure from 1-ethynyl-2-nitrobenzene **1l** (59 mg, 0.4 mmol). Reaction time 20 min. The crude was purified by flash column chromatography (SiO_2, Hex/EtOAc 9:1). **2l** was obtained in 13% yield (9 mg) as a yellow solid. ^{1}H NMR (300 MHz, $CDCl_3$): 8.10 (dd, *J* = 8.1, 1.2 Hz, 1H), 7.72 (td, *J* = 7.5, 1.2 Hz, 1H), 7.60 (ddd, *J* = 8.2, 7.5, 1.5 Hz, 1H), 7.46 – 7.41 (m, 1H), 2.56 (s, 3H). Spectral data are in good agreement with literature values. [15]

1-(2-Isopropylphenyl)ethan-1-one 2m

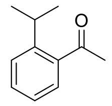

Synthetized following the general procedure from 1-ethynyl-2-isopropylbenzene **1m** (58 mg, 0.4 mmol). Reaction time 20 min. **2m** was obtained in 84% yield (55 mg) as a brown oil. ^{1}H NMR (300 MHz, $CDCl_3$): 7.48 (d, *J* = 7.6 Hz, 1H), 7.42 (d, *J* = 3.7 Hz, 2H), 7.27 – 7.19 (m, 1H), 3.46 (hept, *J* = 6.9 Hz, 1H), 2.57 (s, 3H), 1.24 (d, *J* = 6.8 Hz, 6H). Spectral data are in good agreement with literature values.[16]

1-(Thiophen-3-yl)ethan-1-one 2n

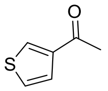

Synthetized following the general procedure from 1-ethynyl-2-fluorobenzene **1n** (43mg, 0.4 mmol). Reaction time 20 min. **2n** was obtained in 100% yield (50 mg) as a yellow oil. ^{1}H NMR (300 MHz, $CDCl_3$): 8.05 (d, *J* = 2.3 Hz, 1H), 7.54 (dd, *J* = 5.1, 1.3 Hz, 1H), 7.32 (dd, *J* = 5.1, 2.9 Hz, 1H), 2.54 (s, 3H). Spectral data are in good agreement with literature values.[17]

1-(Naphthalen-1-yl)ethan-1-one 2o

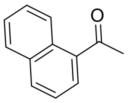

Synthetized following the general procedure from 1-ethynylnaphthalene **1o** (61 mg, 0.4 mmol). Reaction time 20 min. **2o** was obtained in 100% yield (68 mg) as a yellow oil. ^{1}H NMR (300 MHz, $CDCl_3$): 8.48 (s, 1H), 8.07 – 8.01 (m, 1H), 7.97 (d, *J* = 7.9 Hz, 1H), 7.93 – 7.86 (m, 2H), 7.65 – 7.52 (m, 2H), 2.74 (s, 3H). Spectral data are in good agreement with literature values. [9]

General procedures for the synthesis of internal ketones 4a-i

Method A

In a screw capped vial, the appropriate alkyne **3a-i** was added to the selected DES (GA/CSA/H_2O, 0.5 mL), and the mixture was heated at 90 °C in an oil bath. Upon completion, as monitored by TLC, the reaction mixture was diluted with H_2O (20 mL) and extracted with Et_2O (3 × 10 mL). The combined organic layers were washed with saturated $NaHCO_3$ solution (20 mL) and brine (20 mL), dried over Na_2SO_4, filtered, and concentrated under reduced pressure. The crude reaction mixture was purified by flash column chromatography on silica gel to afford the corresponding ketone.

Method B

In a MW vial, the appropriate alkyne **3a-i** was added to the selected DES (GA/CSA/H_2O, 0.5 mL), and the mixture was heated at 100 °C under MW irradiation for the appropriate time. Upon completion, as monitored by TLC, the reaction mixture was diluted with H_2O (20 mL) and extracted with EtOAc (3 × 10 mL). The combined organic layers were washed with saturated $NaHCO_3$ solution (20 mL) and brine (20 mL), dried over Na_2SO_4, filtered, and concentrated under reduced pressure. The crude reaction mixture was purified by flash column chromatography on silica gel to afford the corresponding ketone.

1-(4-Methoxyphenyl)-2-(*p*-tolyl)ethan-1-one 4a

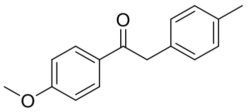

Synthetized from 1-methoxy-4-(*p*-tolylethynyl) benzene **3a** (44 mg, 0.2 mmol). Purification of the crude by flash column chromatography (SiO_2, Hex/EtOAc 9:1) afforded **4d** in 85% yield(41 mg, 60 min) with Method A and in 82% yield(40 mg, 30 min) with Method B, as a pale-yellow solid. ^{1}H NMR (300 MHz, $CDCl_3$): 8.02 – 7.96 (m, 2H), 7.19 – 7.09 (m, 4H), 6.95 – 6.89 (m, 2H), 4.19 (s, 2H), 3.86 (s, 3H), 2.32 (s, 3H). Spectral data are in good agreement with literature values.[18]

2-(4-Fluorophenyl)-1-(*p*-tolyl)ethan-1-one 4b

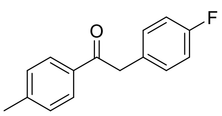

Synthetized from 1-fluoro-4-(*p*-tolylethynyl)benzene **3b** (42 mg, 0.2 mmol). Purification of the crude by flash column chromatography (SiO_2, Hex/EtOAc 98:2 → 95:5) afforded **4b** in 66% yield(30 mg, 240 min) with Method A and in 95% yield(43 mg, 120 min) with Method B, as a white solid. ^{1}H NMR (300 MHz, $CDCl_3$): 8.03 (d, *J* = 8.3 Hz, 2H), 7.43 –

7.29 (m, 4H), 7.17 – 7.06 (m, 2H), 4.36 (s, 2H), 2.54 (s, 3H). Spectral data are in good agreement with literature values.[19]

2-(2-Fluorophenyl)-1-(*p*-tolyl)ethan-1-one 4c

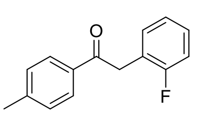

Synthetized from 1-fluoro-2-(*p*-tolylethynyl)benzene **3b** (42 mg, 0.2 mmol). Purification of the crude by flash column chromatography (SiO_2, Hex/EtOAc 98:2) afforded **4b** in 79% yield(36 mg, 240 min) with Method A and in 100% yield(45 mg, 120 min) with Method B, as a pale yellow solid. ^1H NMR (300 MHz, $CDCl_3$): 7.94 (d, J = 8.3 Hz, 2H), 7.25 (dd, J = 13.0, 7.9 Hz, 4H), 7.15 – 7.02 (m, 2H), 4.30 (s, 2H), 2.42 (s, 3H). ^{13}C NMR (75 MHz, $CDCl_3$): 195.97 (C), 160.96 (C, d, J = 245.5 Hz), 144.14 (C), 133.95 (C), 131.62 (CH, d, J = 4.1 Hz), 129.37 (2xCH), 128.81 (CH, d, J = 8.0 Hz), 128.54 (2xCH), 124.14 (CH, d, J = 3.2 Hz), 122.06 (C, d, J = 16.5 Hz), 115.36 (CH, d, J = 22.7 Hz), 38.45 (CH_2), 21.66 (CH_3). ESI(+)-MS: m/z (%) = 229.12 (100) $[M + H]^+$.

1-(*p*-Tolyl)-2-(3-(trifluoromethyl)phenyl)ethan-1-one 4d

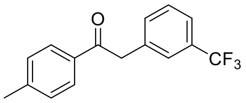

Synthetized from 1-(*p*-tolylethynyl)-3-(trifluoromethyl)benzene **3d** (52 mg, 0.2 mmol). Purification of the crude by flash column chromatography (SiO_2, Hex/EtOAc 98:2) afforded **4d** in 27% yield(15 mg, 390 min) with Method A and in 30% yield(17 mg, 120 min) with Method B, as a white solid. ^1H NMR (300 MHz, $CDCl_3$): 7.92 (d, J = 8.3 Hz, 2H), 7.58 – 7.41 (m, 4H), 7.31 – 7.24 (m, 2H), 4.33 (s, 2H), 2.42 (s, 3H). ^{13}C NMR (75 MHz, $CDCl_3$): 196.25 (C), 144.40 (C), 135.56 (C), 133.86 (C), 133.05 (CH), 130.88 (C, q, J = 32.2 Hz), 129.46 (2xCH), 128.95 (C), 128.59 (2xCH), 126.37 (CH, q, J = 3.7 Hz), 124.06 (C, q, J = 272.1 Hz), 123.76 (CH, q, J = 3.9 Hz), 44.80 (CH_2), 21.66 (CH_3). ESI(+)-MS: m/z (%) = 279.09 (100) $[M + H]^+$.

2-(3-Methoxyphenyl)-1-(*p*-tolyl)ethan-1-one 4e

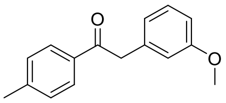

Synthetized from 1-methoxy-3-(*p*-tolylethynyl)benzene **3e** (44 mg, 0.2 mmol). Purification of the crude by flash column chromatography (SiO_2, Hex/EtOAc 95:5) afforded **4b** in 66% yield(32 mg, 120 min) with Method A and in 90% yield(43 mg, 30 min) with Method B, as a yellow liquid. ^1H NMR (300 MHz, $CDCl_3$): 7.94 – 7.87 (m, 2H), 7.28 – 7.18 (m, 3H), 6.89 – 6.75 (m, 3H), 4.22 (s, 2H), 3.78 (s, 3H), 2.40 (s, 3H). Spectral data are in good agreement with literature values.[20]

1-(2-Methoxyphenyl)-2-(*p*-tolyl)ethan-1-one 4f

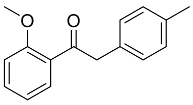

Synthetized from 1-methoxy-2-(*p*-tolylethynyl)benzene **3f** (44 mg, 0.2 mmol). Purification of the crude by flash column chromatography (SiO_2, Hex/EtOAc 98:2 → 95:5) afforded **4f** in 21% yield(10 mg, 90 min) with Method A as a yellow liquid. ^1H NMR (300 MHz, $CDCl_3$): 7.65 (dd, J = 7.7, 1.6 Hz, 1H), 7.44 (ddd, J = 8.3, 7.4, 1.8 Hz, 1H), 7.11 (s, 4H), 7.01 – 6.93 (m, 2H), 4.26 (s, 2H), 3.92 (s, 3H), 2.31 (s, 3H). Spectral data are in good agreement with literature values.[21]

2-(4-Chlorophenyl)-1-(*p*-tolyl)ethan-1-one 4g

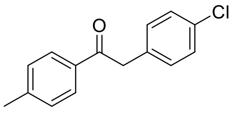

Synthetized from 1-chloro-4-(*p*-tolylethynyl)benzene **3g** (45 mg, 0.2 mmol). Purification of the crude by flash column chromatography (SiO_2, Hex/EtOAc 99:1) afforded **4g** in 71% yield(35 mg, 120 min) with Method B as a white solid. ^1H NMR (300 MHz, $CDCl_3$): 7.89 (d, J = 8.2 Hz, 2H), 7.32 – 7.24 (m, 4H), 7.22 – 7.16 (m, 2H), 4.23 (s, 2H), 2.41 (s, 3H). Spectral data are in good agreement with literature values.[18]

2-Cyclopentyl-1-(*p*-tolyl)ethan-1-one 4h

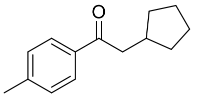

Synthetized from 1-(cyclopentylethynyl)-4-methylbenzene **3h** (37 mg, 0.2 mmol). Purification of the crude by flash column chromatography (SiO_2, Hex/EtOAc 98:2) afforded **4h** in 91% yield(37 mg, 180 min) with Method A and in 73% yield(30 mg, 90 min) with Method B, as a white solid. ^1H NMR (300 MHz, $CDCl_3$): 7.89 – 7.82 (m, 2H), 7.28 – 7.18 (m, 2H), 2.95 (d, *J* = 7.1 Hz, 2H), 2.40 (s, 3H), 1.95 – 1.79 (m, 2H), 1.72 – 1.47 (m, 4H), 1.28 – 1.07 (m, 2H). Spectral data are in good agreement with literature values.[22]

1-(Thiophen-3-yl)-2-(*p*-tolyl)ethan-1-one 4i

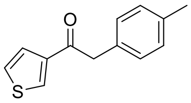

Synthetized from 3-(*p*-tolylethynyl)thiophene **3i** (40 mg, 0.2 mmol). Purification of the crude by flash column chromatography (SiO_2, Hex/EtOAc 99:1→ 95:5) afforded **4i** in 60% yield(26 mg, 120 min) with Method A and in 80% yield(35 mg, 90 min) with Method B, as a white solid. ^1H NMR (300 MHz, $CDCl_3$): 8.11 (dd, *J* = 2.9, 1.3 Hz, 1H), 7.59 (dd, *J* = 5.1, 1.3 Hz, 1H), 7.32 (dd, *J* = 5.1, 2.9 Hz, 1H), 7.21 – 7.13 (m, 4H), 4.16 (s, 2H), 2.35 (s, 3H). ^{13}C NMR (101 MHz, $CDCl_3$): 192.10 (C), 141.89 (C), 136.59 (C), 132.60 (CH), 131.36 (C), 129.43 (2xCH), 129.24 (2xCH), 127.38 (CH), 126.30 (CH), 46.60 (CH_2), 21.08 (CH_3). ESI(+)-MS: m/z (%) = 217.41 (100) $[M + H]^+$.

2-(Thiophen-3-yl)-1-(*p*-tolyl)ethan-1-one 4i'

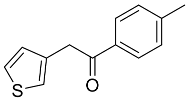

Synthetized from 3-(*p*-tolylethynyl)thiophene **3i** (40 mg, 0.2 mmol). Purification of the crude by flash column chromatography (SiO_2, Hex/EtOAc 99:1 → 95:5) afforded **4i'** in 10% yieldyield (5 mg, 120 min) with Method A and in 10% yieldyield (4 mg, 90 min) with Method B, as a white solid. ^1H NMR (300 MHz, $CDCl_3$): 7.91 (d, *J* = 8.2 Hz, 2H), 7.33 – 7.21 (m, 4H), 7.12 (s, 1H), 7.02 (d, *J* = 5.0 Hz, 1H), 4.28 (s, 2H), 2.41 (s, 3H). ^{13}C NMR (75 MHz, $CDCl_3$): 196.74 (C), 144.05 (C), 134.38 (C), 133.99 (C), 129.34 (2xCH), 128.70 (2xCH), 128.63 (CH), 125.71 (CH), 122.71 (CH), 39.97 (CH_2), 21.65 (CH_3). ESI(+)-MS: m/z (%) = 217.41 (100) $[M + H]^+$.

Recycle trial for the synthesis of 2c

In a MW vial, the alkyne **1c** (2 mmol, 240 mg) was added to the selected DES (GA/pTSA·H_2O/H_2O, 5 mL), and the mixture was heated at 100 °C under MW irradiation for 20 min. Upon completion, the reaction mixture was diluted with deionized H_2O (20 mL) and extracted with Et_2O (2 × 10 mL). The combined organic layers were washed with saturated $NaHCO_3$ solution (20 mL) and brine (20 mL), dried over Na_2SO_4, filtered, and concentrated under reduced pressure to afford the corresponding ketone **2c**. The aqueous phase was evaporated under reduced pressure to restore the initial volume of the DES. Variations in the water content of the DES are not critical, as it has been demonstrated that the eutectic nature of the mixture is maintained over a relatively wide range of water concentrations.[3] The DES was then used for a new cycle.

Recycle trial for the synthesis of 1-(4-methoxyphenyl)-2-(*p*-tolyl)ethan-1-one 4a

In a screw capped vial, the alkyne **2a** (1 mmol, 222 mg) was added to the selected DES (GA/CSA/H_2O, 5 mL), and the mixture was heated at 90 °C in an oil bath. Upon completion, as monitored by TLC, the reaction mixture was diluted with deionized H_2O (20 mL) and extracted with EtOAc (2 × 10 mL). The combined organic layers were washed with saturated $NaHCO_3$ solution (20 mL) and brine (20 mL), dried

over Na_2SO_4, filtered, and concentrated under reduced pressure to afford the corresponding ketone **4a**. The aqueous phase was evaporated under reduced pressure to restore the initial volume of the DES. Variations in the water content of the DES are not critical, as it has been demonstrated that the eutectic nature of the mixture is maintained over a relatively wide range of water concentrations.[3] The DES was then used for a new cycle.

Simple E-Factor (sEF)[23] calculation for the synthesis of acetophenone 2a.

Reagents = 670 mg (0.5 mL GA/PTSA·H_2O/H_2O) + 41 mg (phenylacetylene **1a**) = 711 mg
Final product **2a** = 48 mg
Waste = 711 mg – 48 mg = 663 mg
sEF = 663 mg / 48 mg = **13.81**

Simple E-Factor (sEF)[23] calculation for the synthesis of 1-(4-methoxyphenyl)-2-(*p*-tolyl)ethan-1-one 4a.
Reagents = 680 mg (0.5 mL GA/CSA/H_2O) + 44 mg (1-methoxy-4-(*p*-tolylethynyl)benzene **3a**) = 724 mg
Final product **4a** = 41 mg
Waste = 724 mg – 41 mg = 683 mg
sEF = 683 mg / 41 mg = **16.66**

Simple E-Factor (sEF)[23] calculation for the synthesis of 1-(4-fluorophenyl)propan-1-one 2c (4 re-cycles)**.**
Reagents = 6700 mg (5 mL GA/PTSA·H_2O/H_2O) + 788 mg (*p*-fluorophenylacetylene **1c**) = 7488 mg
Final product **2c** = 896 mg
Waste = 7488 mg – 896 mg = 6592 mg
sEF = 6592 mg / 896 mg = **7.36**

Simple E-Factor (sEF)[23] calculation for the synthesis of 1-(4-methoxyphenyl)-2-(*p*-tolyl)ethan-1-one 4a (4 re-cycles)**.**
Reagents = 6800 mg (5 mL GA/CSA/H_2O) + 659 mg (1-methoxy-4-(*p*-tolylethynyl)benzene **3a**) = 7459 mg
Final product **2c** = 685 mg
Waste = 7459 mg – 685 mg = 6774 mg
sEF = 6774 mg / 685 mg = **9.89**

EcoScale[24] calculation for the MW-promoted synthesis acetophenone 2a.

Parameters	For 2a synthesis	Penalty points
**1.** Yield (100 – % yield)/2	100-100/2 = 0	0
**2.** Price of reaction components (to obtain 10 mmol of product)		
Inexpensive (< $10)	[PTSA, GA, H_2O, Ph-C≡CH]	0
**3.** Safety		
F (Highly flammable)	[Ph-C≡CH]	5
**4.** Technical Set-up		
Unconventional activation technique	[Microwaves]	2
**5.** Temperature/time		
Heating, < 1 h	[100 °C, 20 min]	2
**6.** Work-up and Purification		
Cooling to room temperature		0
Adding solvent		0
Liquid-liquid extraction		3
Removal of solvent with bp < 150°C		0
**Starting score**		**100**
**Penalty Points Total**		**12**
**EcoScale**		**88**
**EcoScale ranking:** > 75, excellent; > 50, acceptable; and < 50, inadequate.		

EcoScale[24] calculation for the synthesis 1-(4-methoxyphenyl)-2-(*p*-tolyl)ethan-1-one 4a.

Parameters	For 4a synthesis	Penalty points
**1.** Yield (100 – % yield)/2	100-85/2 = 0	7.5
**2.** Price of reaction components (to obtain 10 mmol of product)		
Inexpensive (< $10)	[CSA, GA, H_2O]	0
Expensive (> $10 and < $50)	[4-Me-Ph-C≡C-Ph-4OMe] estimated; this reagent was prepared in the lab	3
**3.** Safety		
		0
**4.** Technical Set-up		
Common setup		0
**5.** Temperature/time		
Heating, < 1 h	[90°C, 60 min]	2
**6.** Work-up and Purification		
Cooling to room temperature		0
Adding solvent		0
Liquid-liquid extraction		3
Removal of solvent with bp < 150°C		0
Chromatography		10
**Starting score**		**100**
**Penalty Points Total**		**25.5**
**EcoScale**		**74.5**
**EcoScale ranking:** > 75, excellent; > 50, acceptable; and < 50, inadequate.		

References

[1] J. Gicquiaud, B. Abadie, K. Dhara, M. Berlande, P. Hermange, J.-M. Sotiropoulos, and P. Y. Toullec, *Chem. Eur. J.* **2020**, *26*, 16266.

[2] (a) Z. Wang, X. Wang, H. Sun, Z. Zhu, G. Zhang, W. Zhang, and Z. Gao, *ChemistrySelect* **2016**, *1*, 391; (b) M. Gholinejad, N. Jeddi, B. Pullithadathil, *Tetrahedron* **2016**, *72*, 2491; (c) R. Hudson, N. P. Bizier, K. N. Esdalea, and J. L. Katz, *Org. Biomol. Chem.* **2015**, *13*, 2273; (d) R. M. Gay, F. Manarin, C. C. Schneider, D. A. Barancelli, M. D. Costa, and G. Zeni, *J. Org. Chem.* **2010**, *75*, 5701; (e) H. Yan, L. Lu, Y. Zhu, H. Yang, D. Liu, G. Rong, and J. Mao, *RSC Adv.* **2013**, *3*, 377.

[3] A. Torregrosa-Chinillach, A. Gritti, E. Brambilla, D. Del Grosso, E. Lepore, D. A. Alonso, R. Chinchilla, G. Abbiati, and M. Tiecco, *J. Mol. Liq.* **2025**, 127110.

[4] A. P. Thankachan, T. G. Abi, K. S. Sindhu, and G. Anilkumar, *ChemistrySelect* **2016**, *1*, 3405.

[5] K. Watanabe, T. Mino, E. Ishikawa, M. Okano, T. Ikematsu, Y. Yoshida, M. Sakamoto, K. Sato, and K. Yoshida, *Eur. J. Org. Chem.* **2017**, 2359.

[6] C. Ye, Y. Li, and H. Bao, *Adv. Synth. Catal.* **2017**, *359*, 3720.

[7] X.-B. Shao, X. Jiang, Q.-H. Li, and Z.-G. Zhao, *Tetrahedron* **2018**, *74*, 6063.

[8] Y. Zhang, Q. Tao, Q. Li, Z. Jin, Y. Long, and X. Zhou, *Chem. Asian J.* **2025**, *20*, e202401768.

[9] Y. Duan, K. Liang, H. Yin, and F.-X. Chen, *Eur. J. Org. Chem.* **2022**, e202200359.

[10] B. Skillinghaug, C. Sköld, J. Rydfjord,F. Svensson, M. Behrends, J. Savmarker, P.J.R. Sjöberg, and M. Larhed, *J. Org. Chem.* **2014**, *79*, 12018.

[11] Z.-W. Chen, D.-N. Ye, Y.-P. Qian, M. Ye, and L.-X. Liu, *Tetrahedron* **2013**, *69,* 6116.

[12] Z. Hyder, J. Ruan, and J. Xiao, *Chem. Eur. J.* **2008**, *14*, 5555.

[13] T. D. Senecal, A. T. Parsons, and S. L. Buchwald, *J. Org. Chem.* **2011**, *76*, 1174.

[14] M. Liu, Z. Hyder, Y. Sun, W. Tang, L. Xu, and J. Xiao, *Org. Biomol. Chem.* **2010**, *8*, 2012.

[15] Y. Yang, A. Qin, K. Zhao, D. Wang, and X. Shi, *Adv. Synth. Catal.* **2016**, *358*, 1433.

[16]K. Gao, and N. Yoshikai, *J. Am. Chem. Soc.* **2013**, *135*, 9279.

[17] S. Saha, A. Sarbajna, and J. K. Bera, *Tetrahedron Lett.* **2014**, *55*, 1444.

[18] S. Xie, Y. Yin, Y. Wang, J. Wang, X. He, R. Bai and R. Shi, *Green Chem.* **2023**, 1522.

[19] J. Liu, X. Zhou, H. Rao, F. Xiao, C.-J. Li, and G.-J. Deng, *Chem. Eur. J.* **2011**, 7996.

[20] C.-S. Kuai, B.-H. Teng, Y. Zhao, and X.-F. Wu, *J. Catal.* **2023**, *425*, 196.

[21] G. Le Bras, O. Provot, J.-F. Peyrat, M. Alami, and J.-D. Brion, *Tetrahedron Lett.* **2006**, *47*, 5497.

[22] W. Deng, C. Ye, Y. Li, and D. Li, *Org. Lett.* **2019**, *21*, 261.

[23] (a) F. Roschangar, R. A. Sheldon, and C. H. Senanayakea, *Green Chem.* **2015**, *17*, 752; (b) R. A. Sheldon, *Green Chem.* **2023**, *25*, 1704.

[24] K. Van Aken, L. Strekowski, and L. Patiny, *Beilstein J. Org. Chem.* **2006**, *2*, 3.

^{1}H NMR (300 MHz, $CDCl_3$)

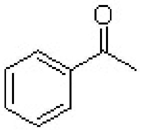

2a

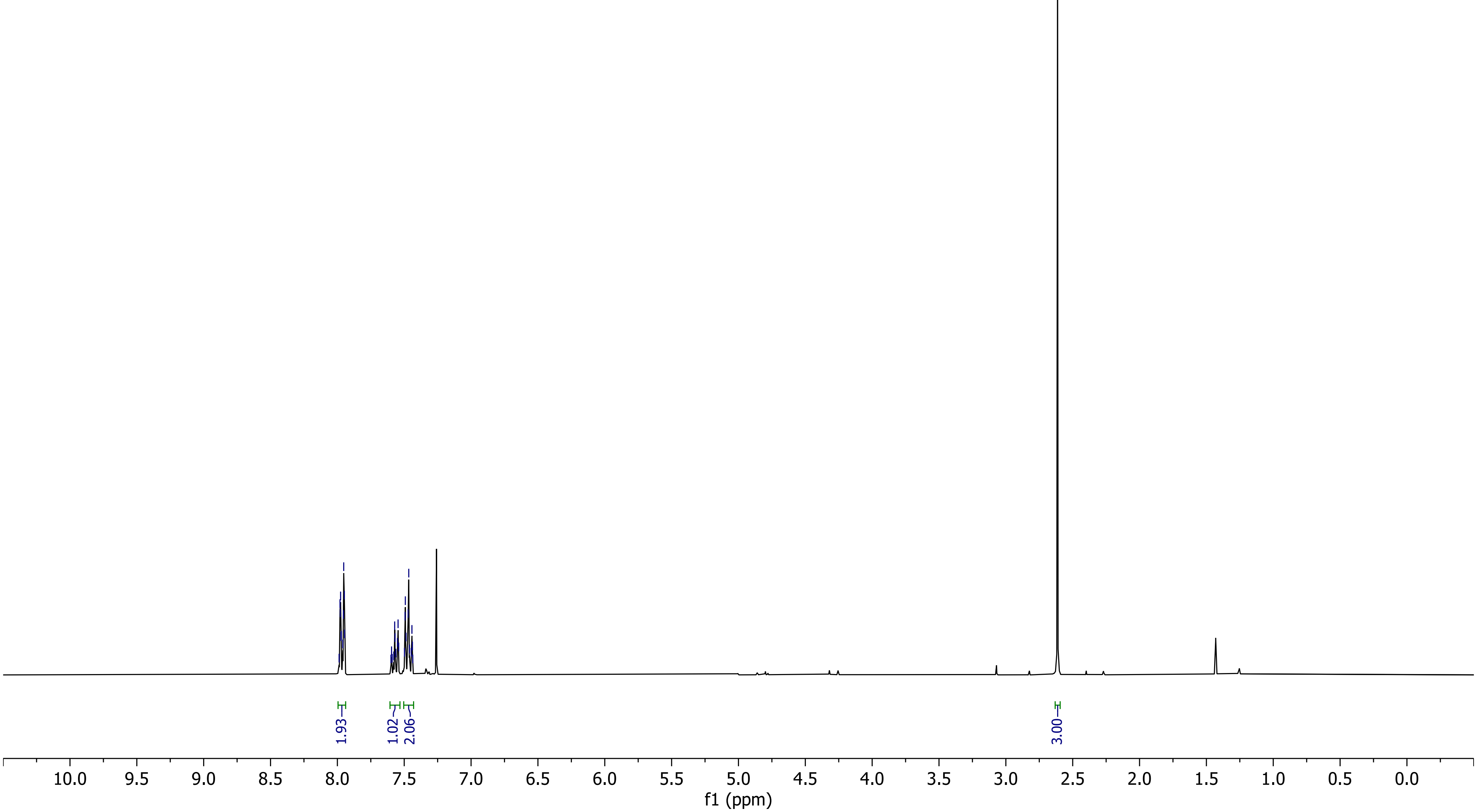

^{1}H NMR (300 MHz, $CDCl_3$)

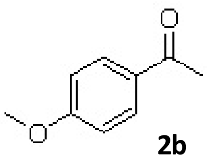

2b

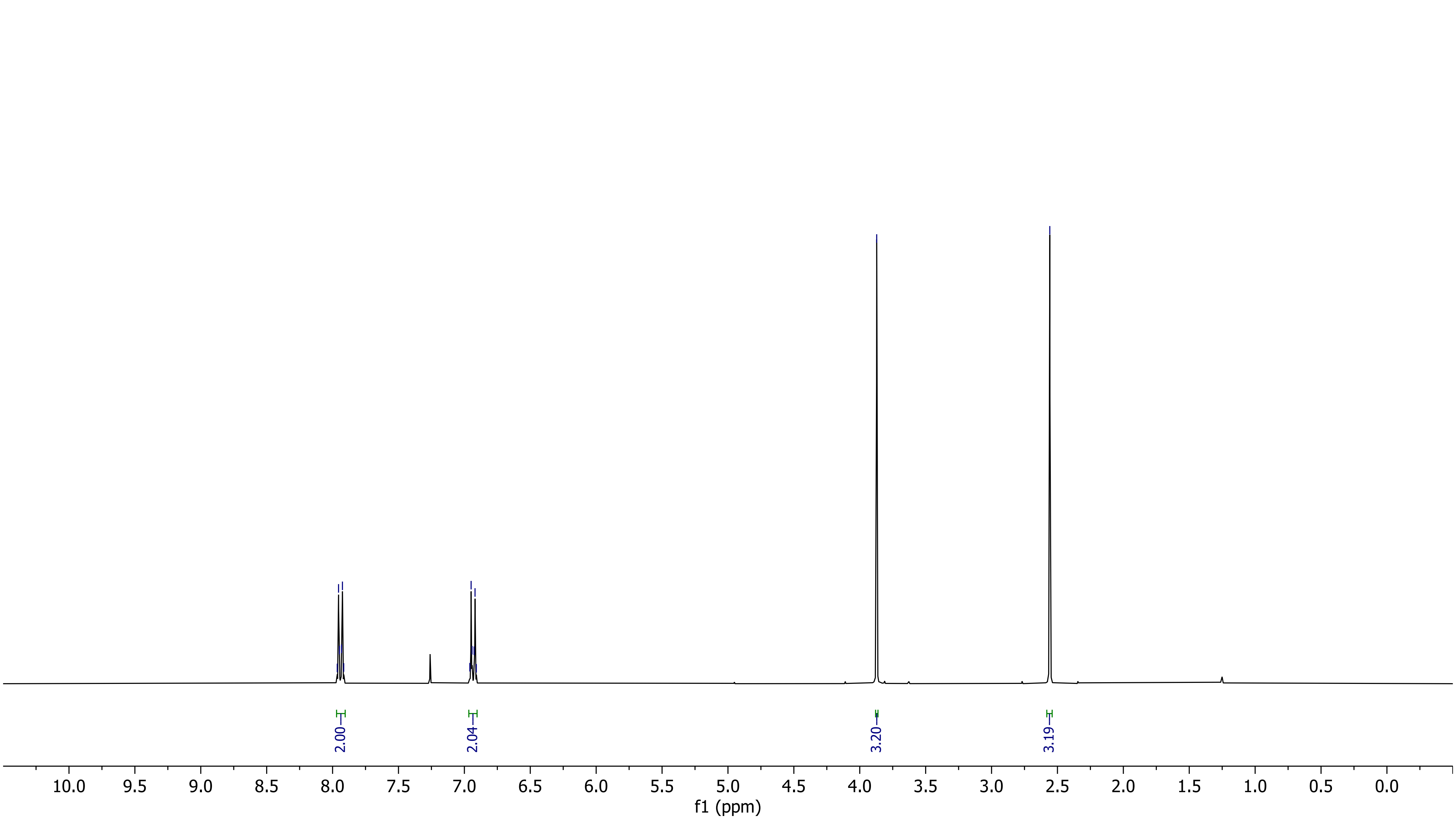

^{1}H NMR (300 MHz, $CDCl_3$)

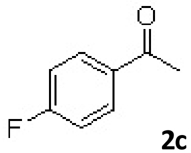

2c

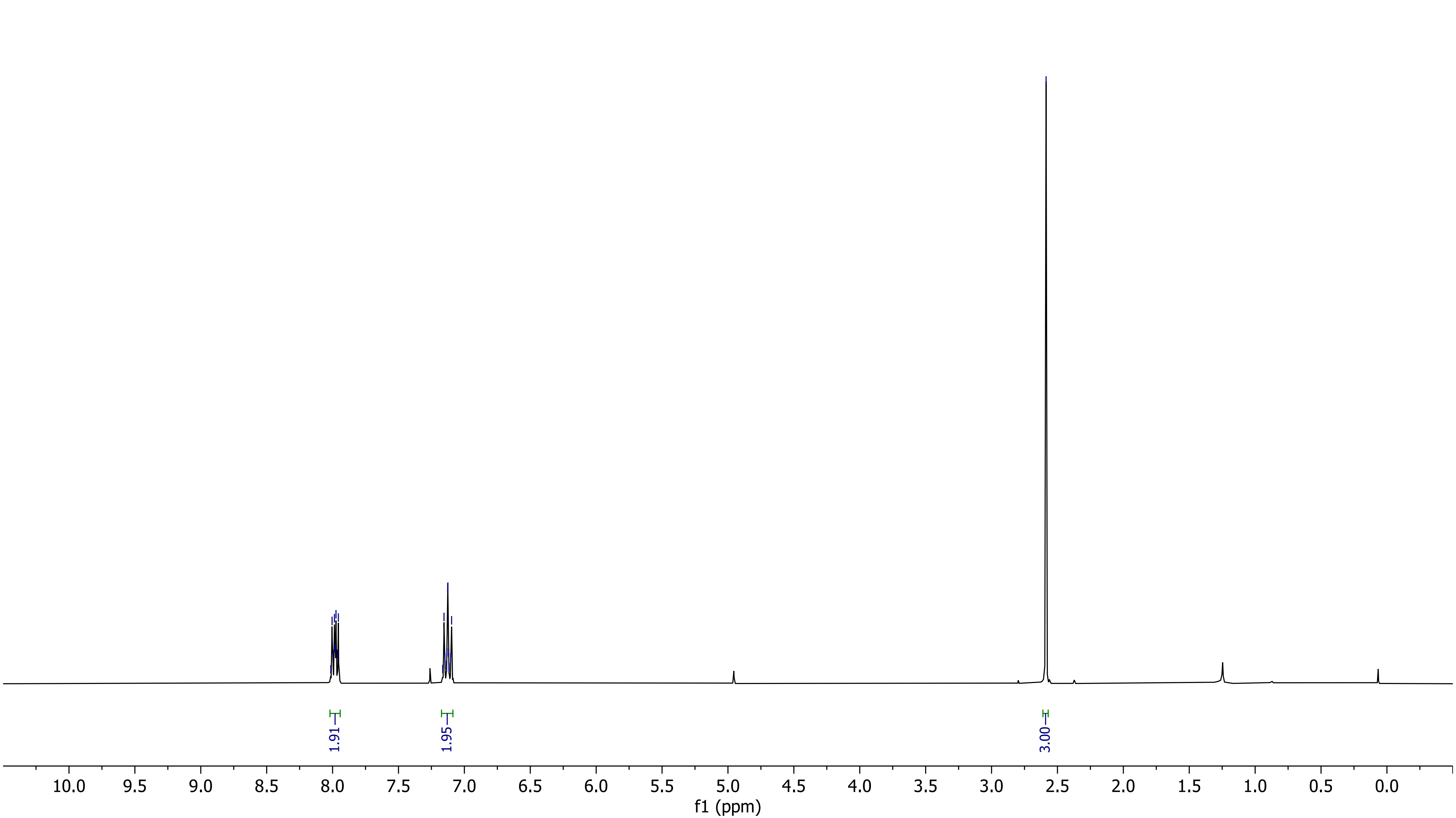

^1H NMR (300 MHz, $CDCl_3$)

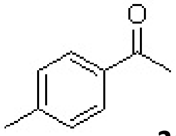

2d

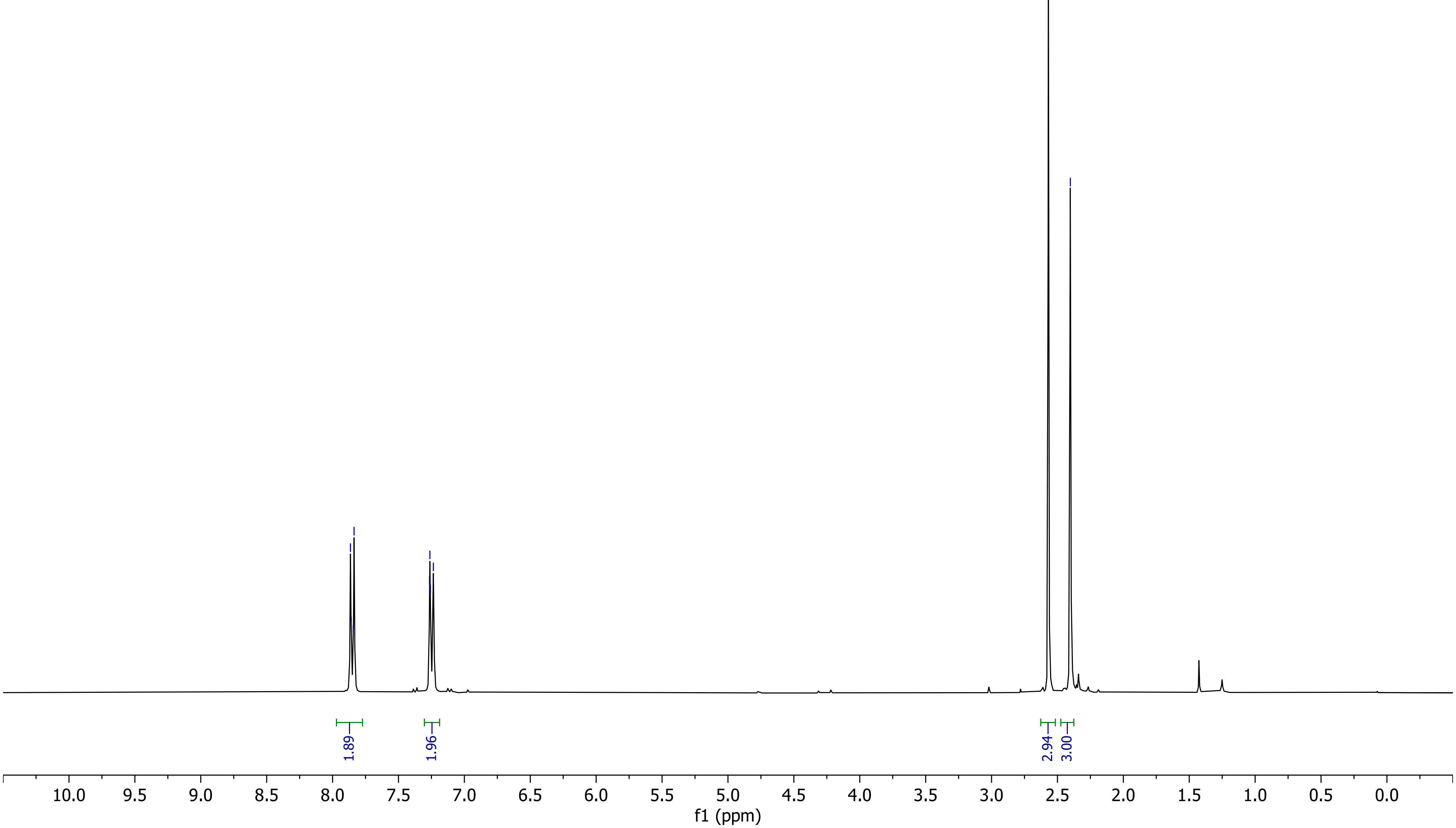

^{1}H NMR (300 MHz, $CDCl_3$)

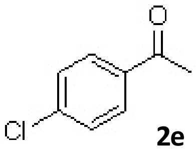

2e

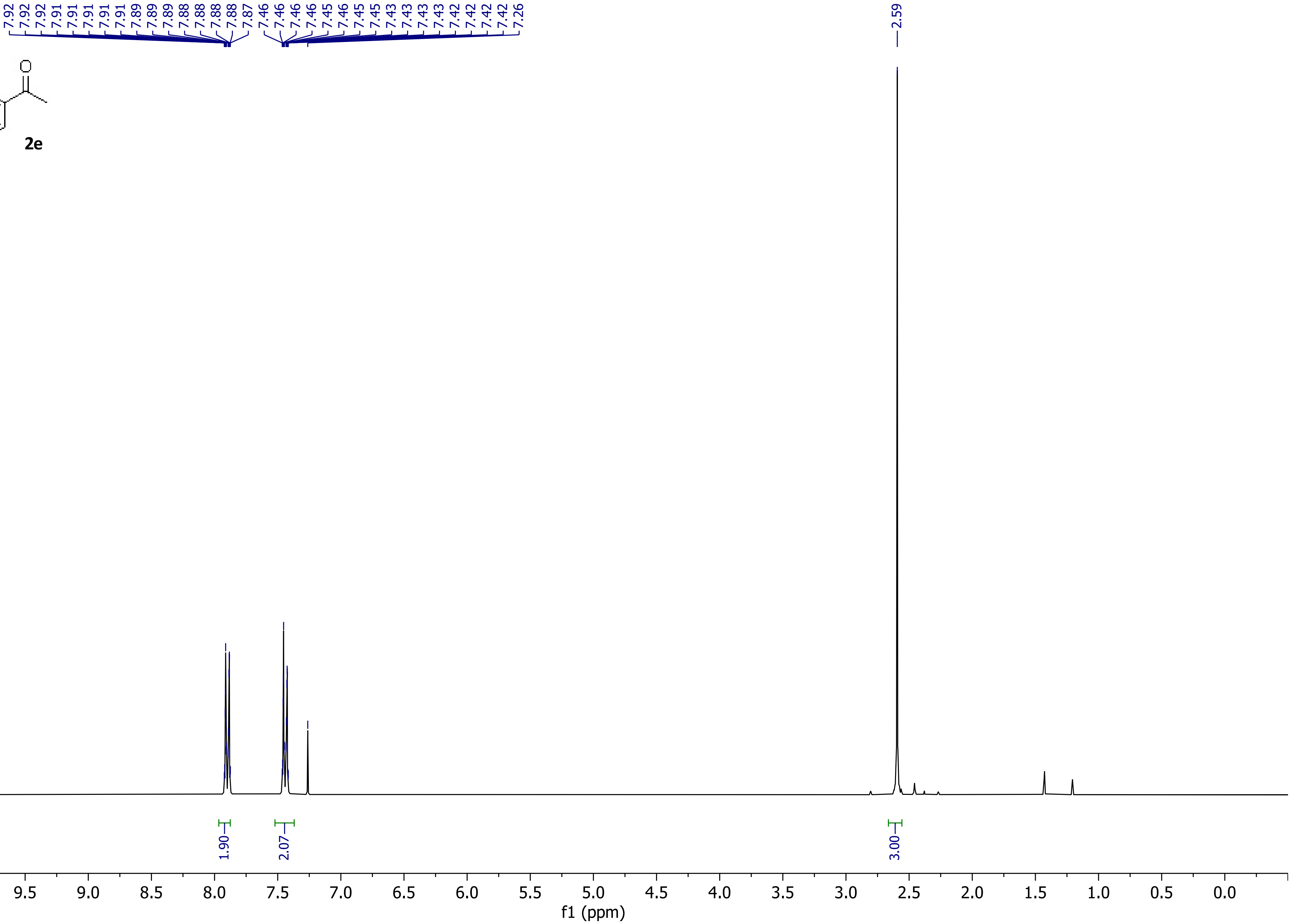

^1H NMR (300 MHz, $CDCl_3$)

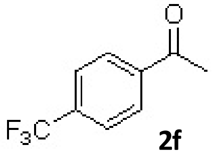

2f

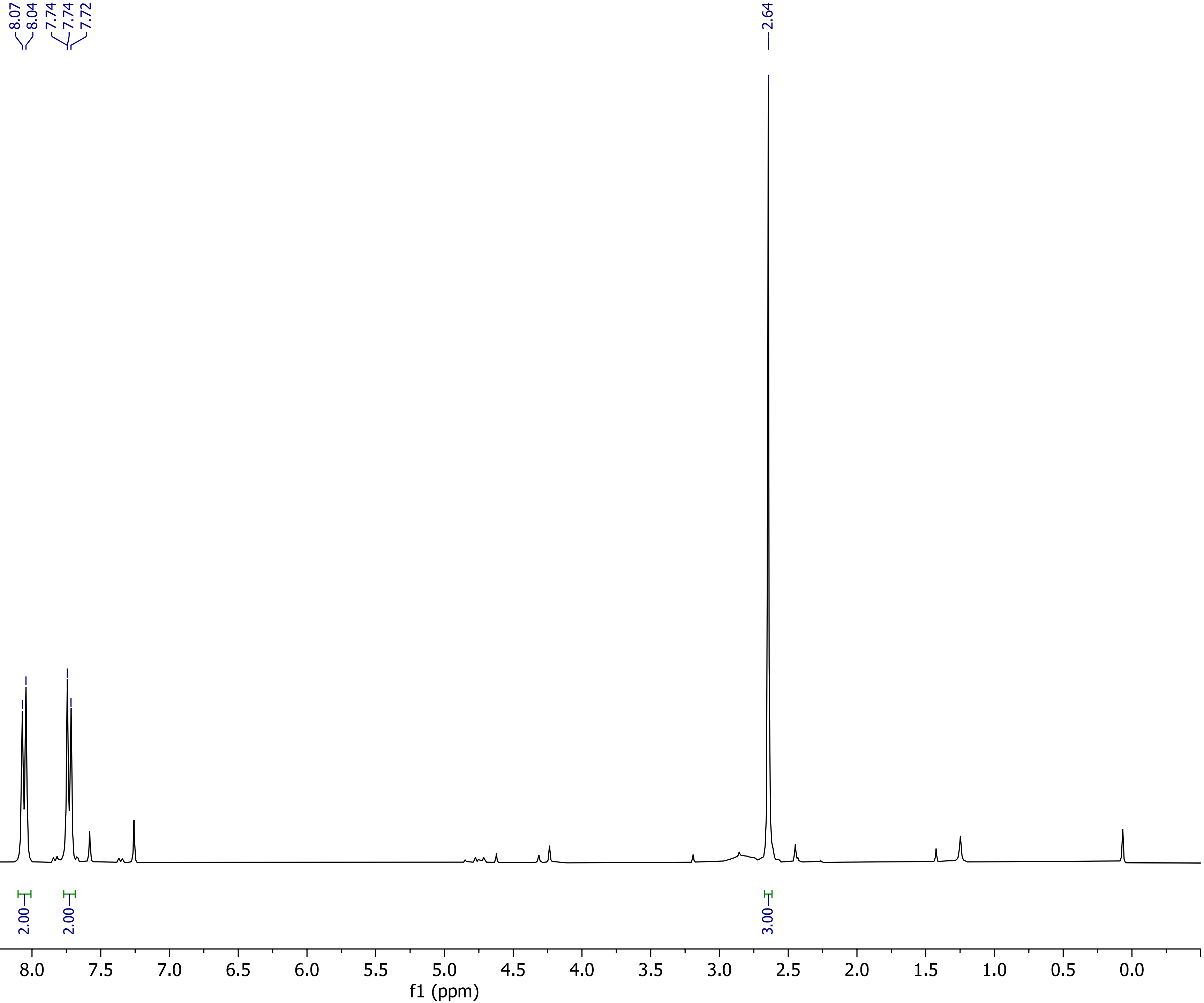

^{1}H NMR (300 MHz, $CDCl_3$)

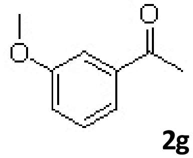

2g

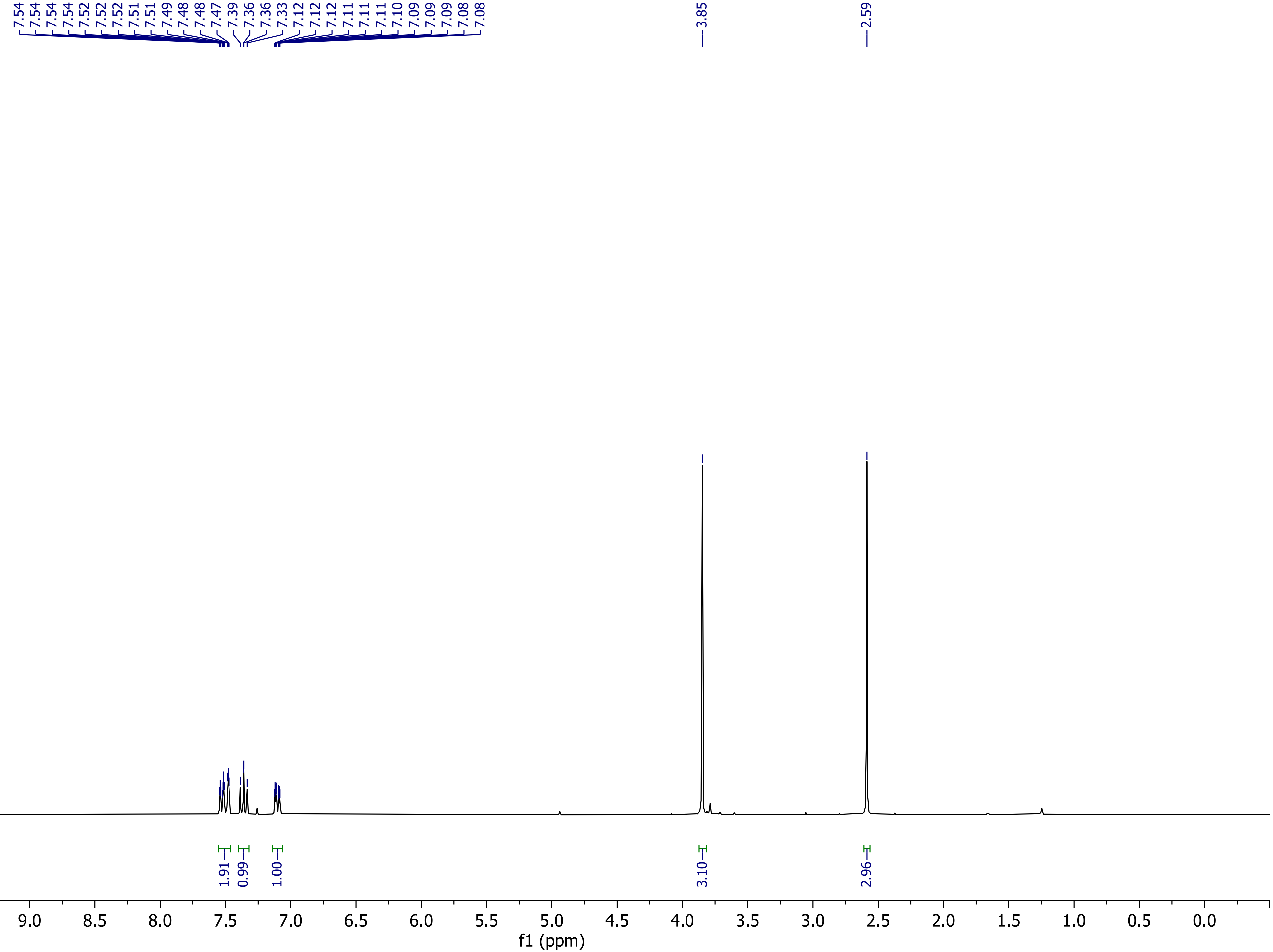

^{1}H NMR (300 MHz, $CDCl_3$)

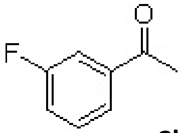

2h

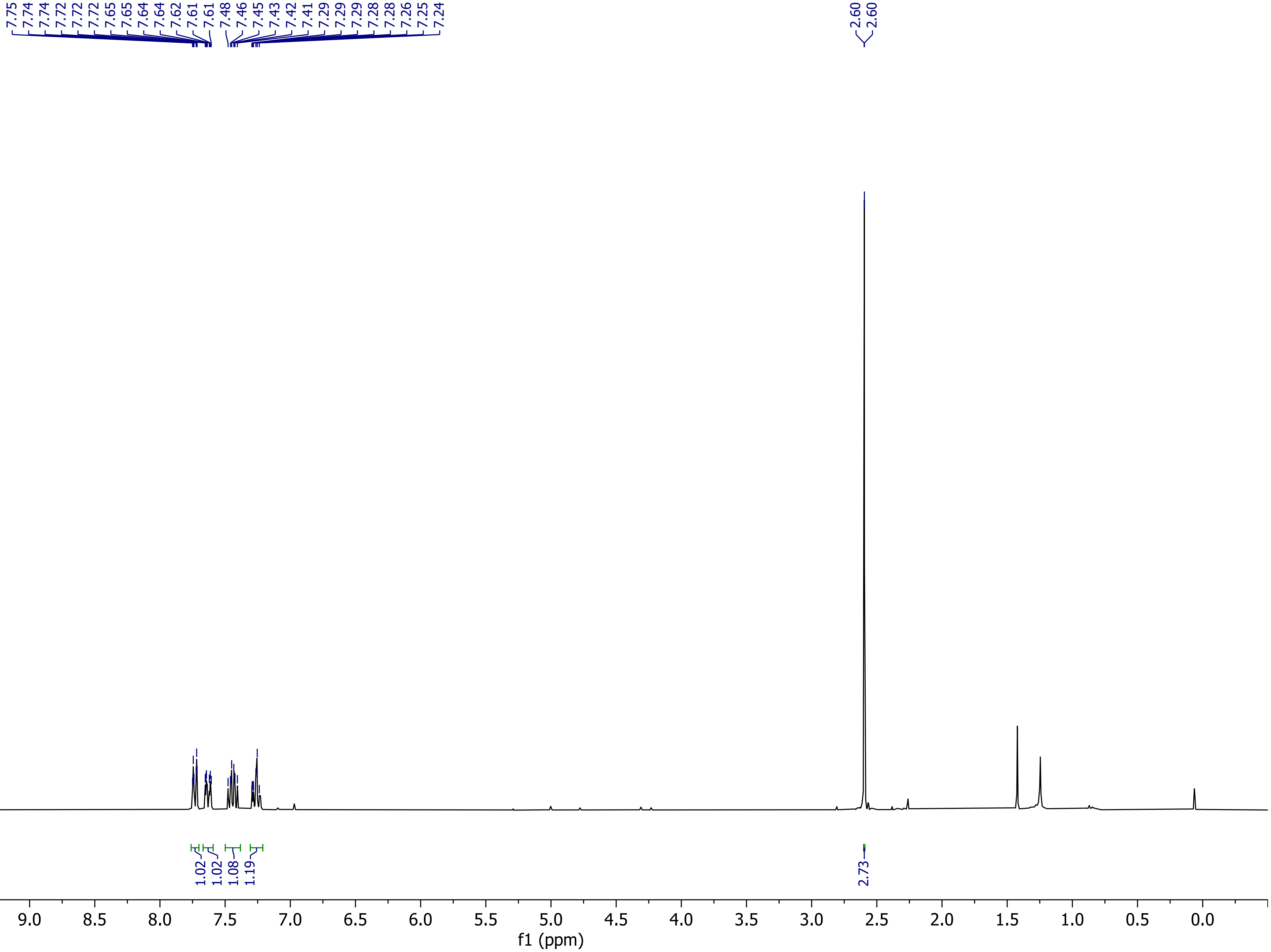

^{1}H NMR (300 MHz, $CDCl_3$)

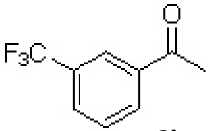

2i

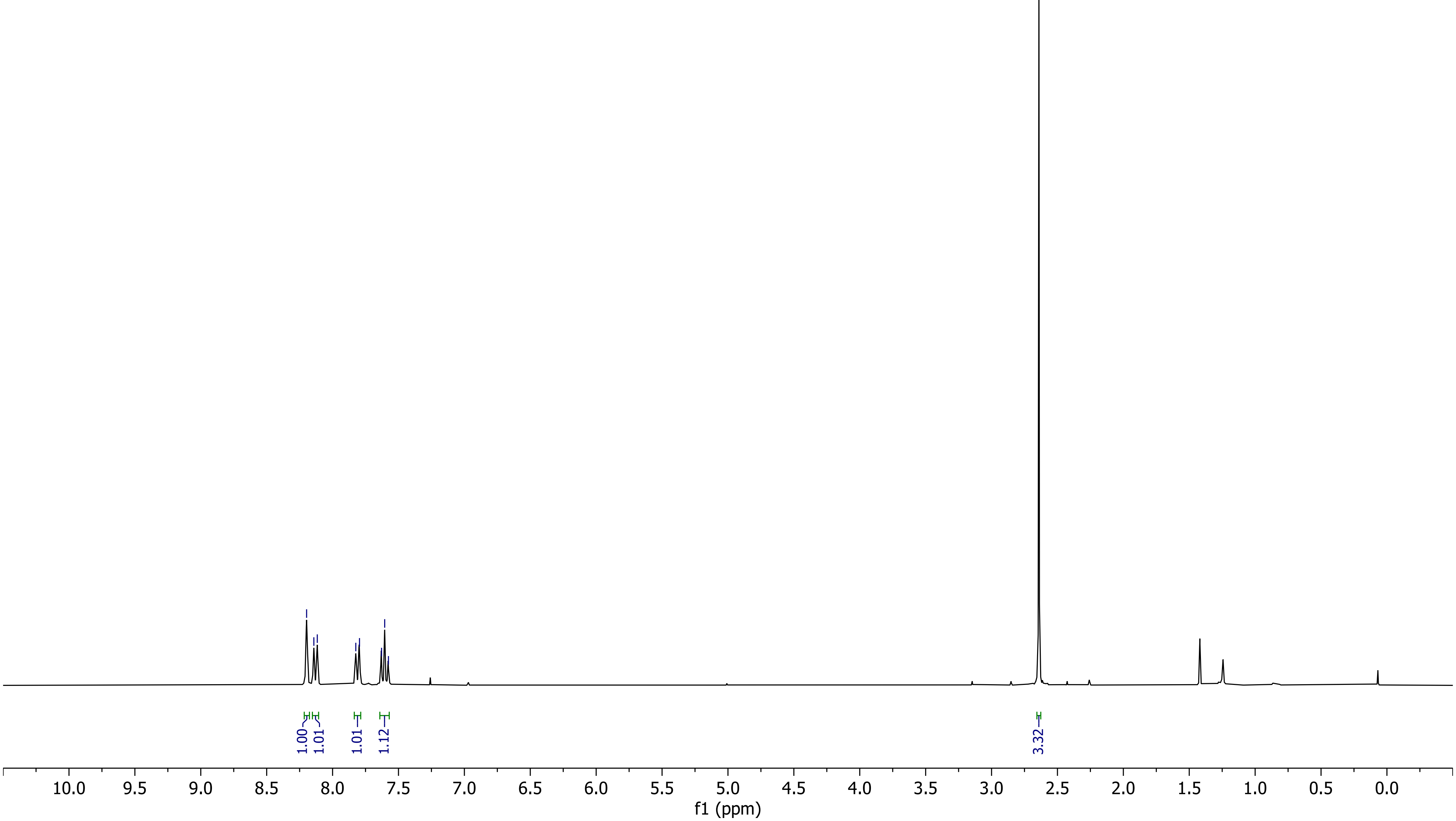

^{1}H NMR (300 MHz, $CDCl_3$)

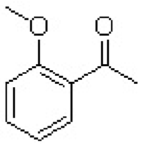

2j

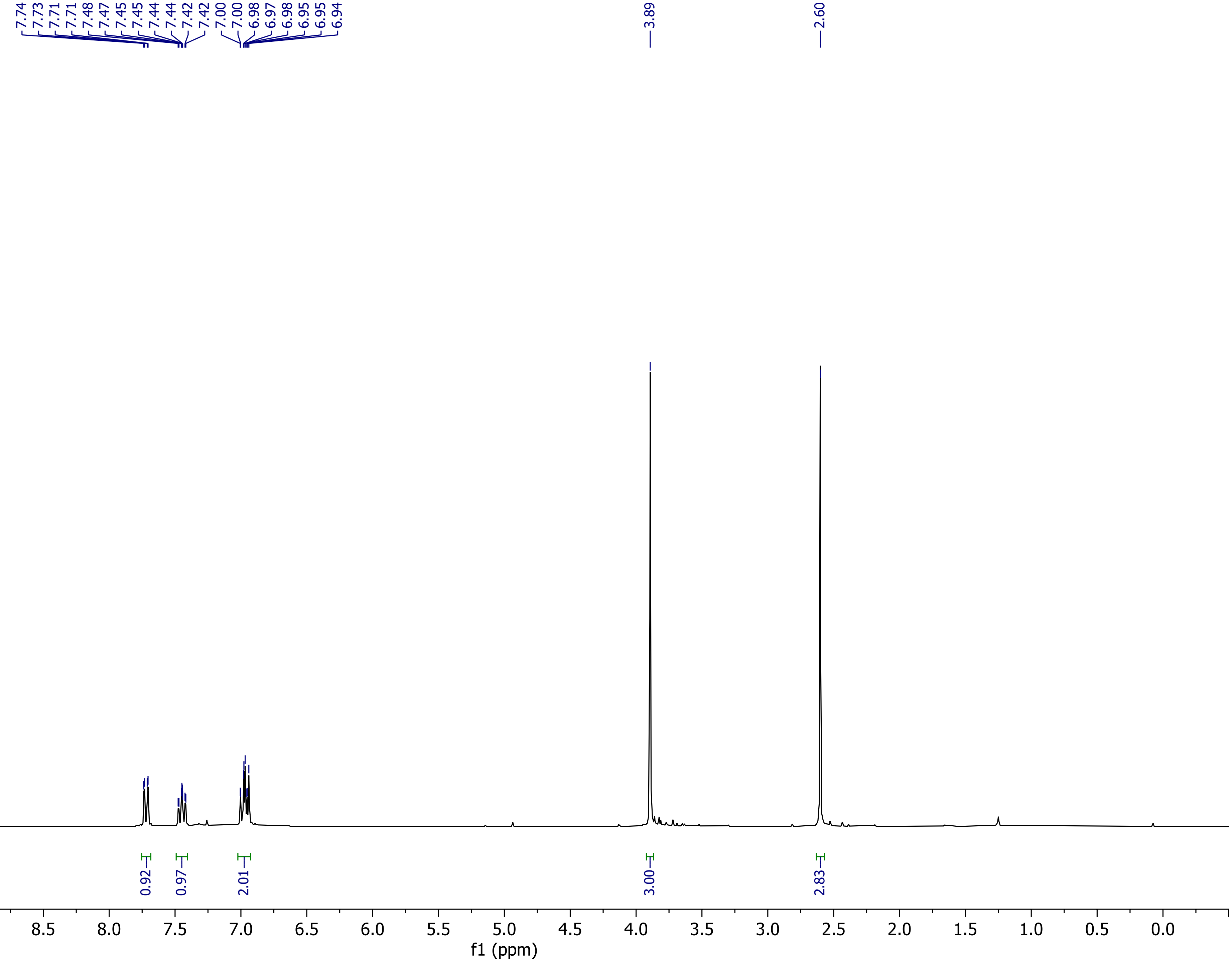

^1H NMR (300 MHz, $CDCl_3$)

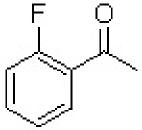

2k

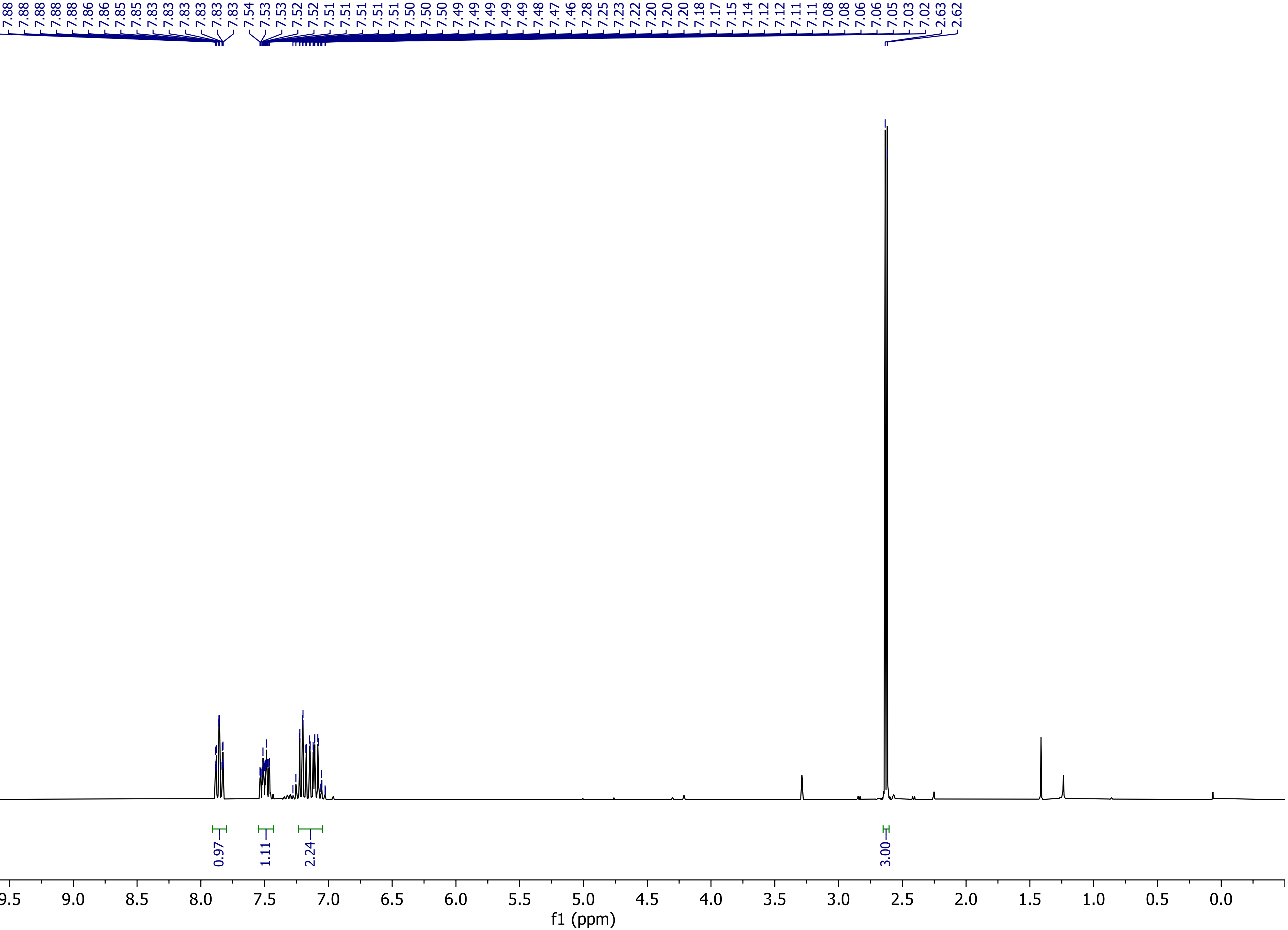

^{1}H NMR (300 MHz, $CDCl_3$)

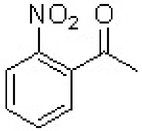

2l

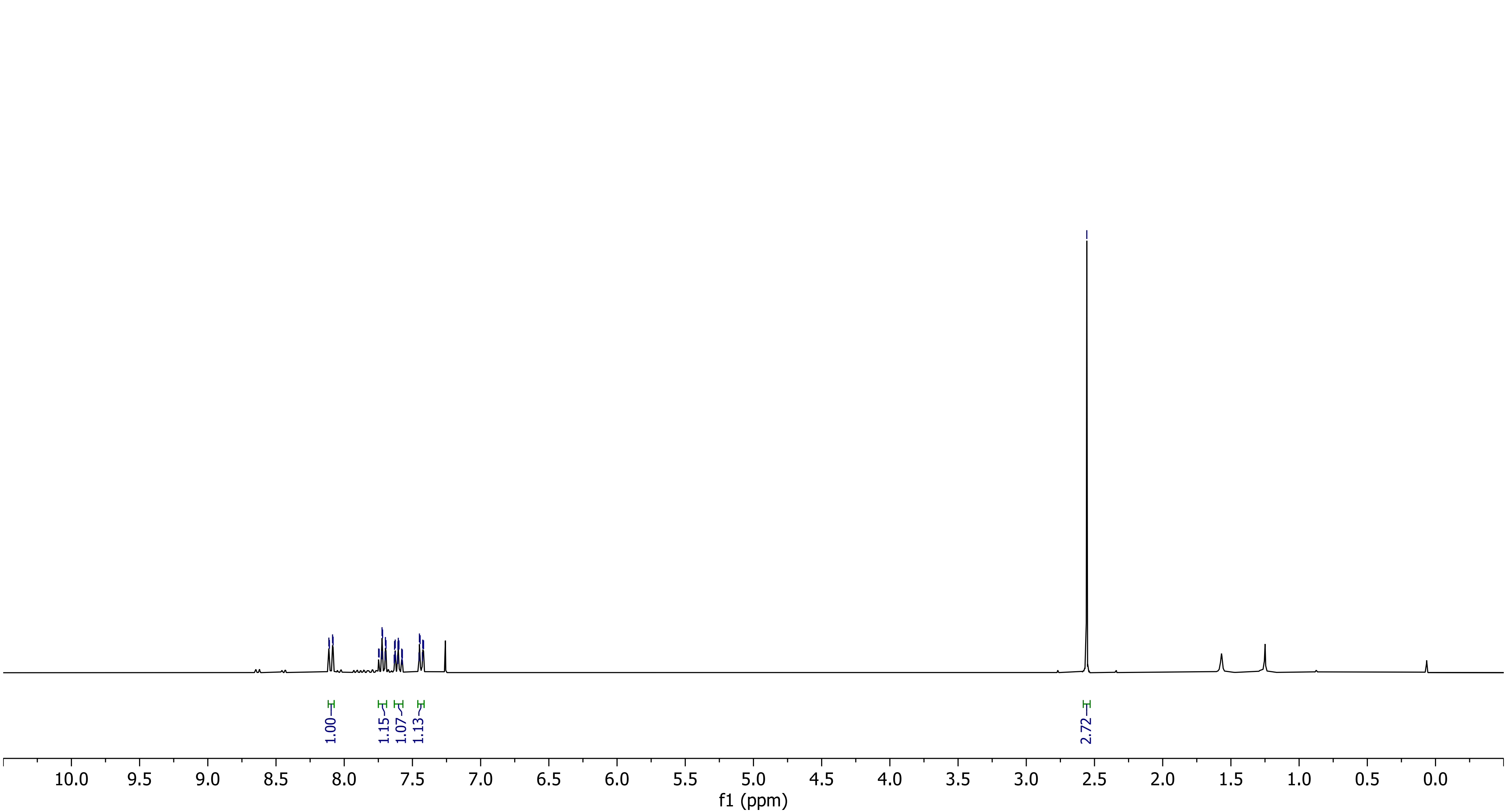

^{1}H NMR (300 MHz, $CDCl_3$)

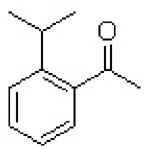

2m

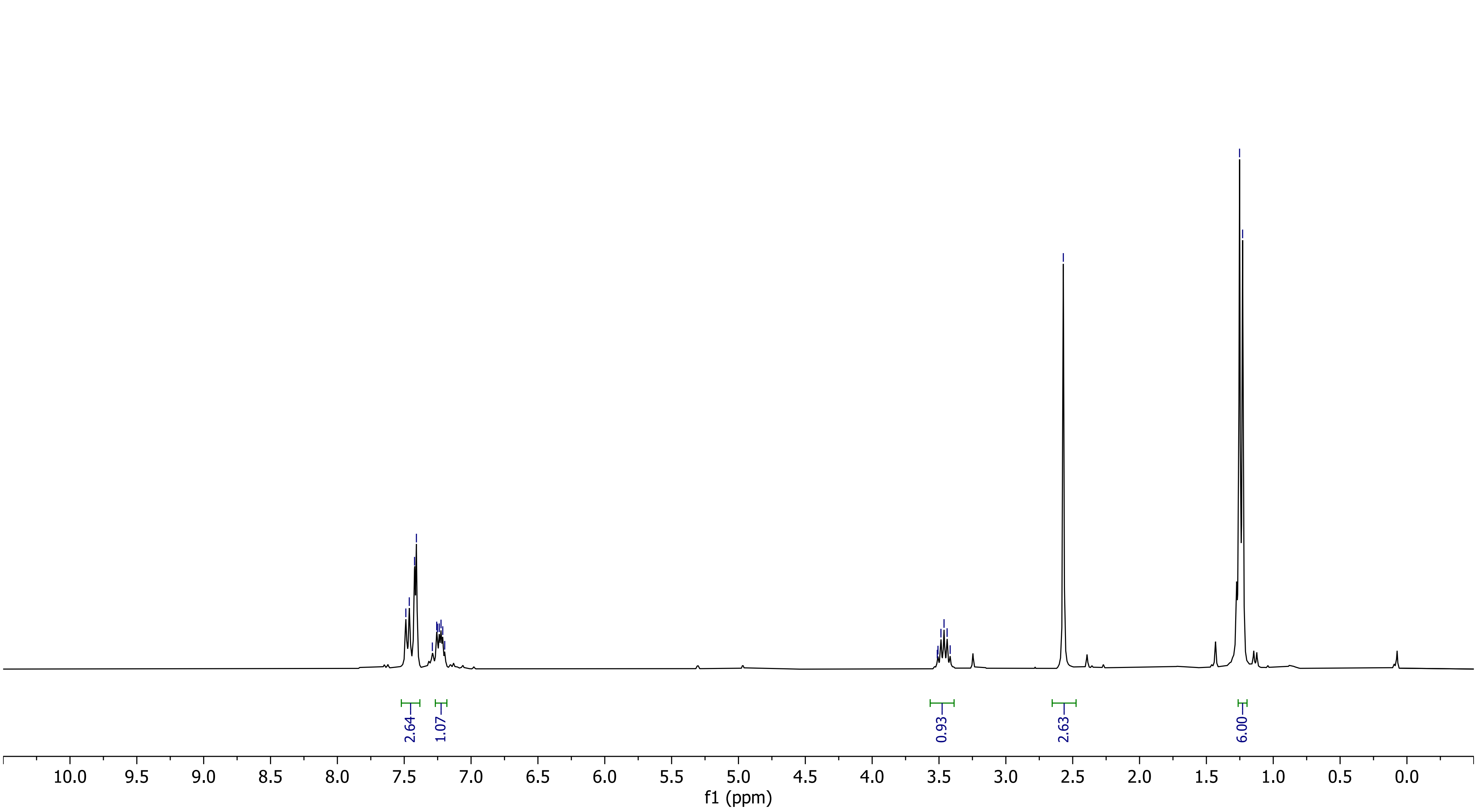

^{1}H NMR (300 MHz, $CDCl_3$)

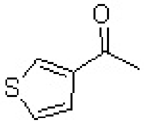

2n

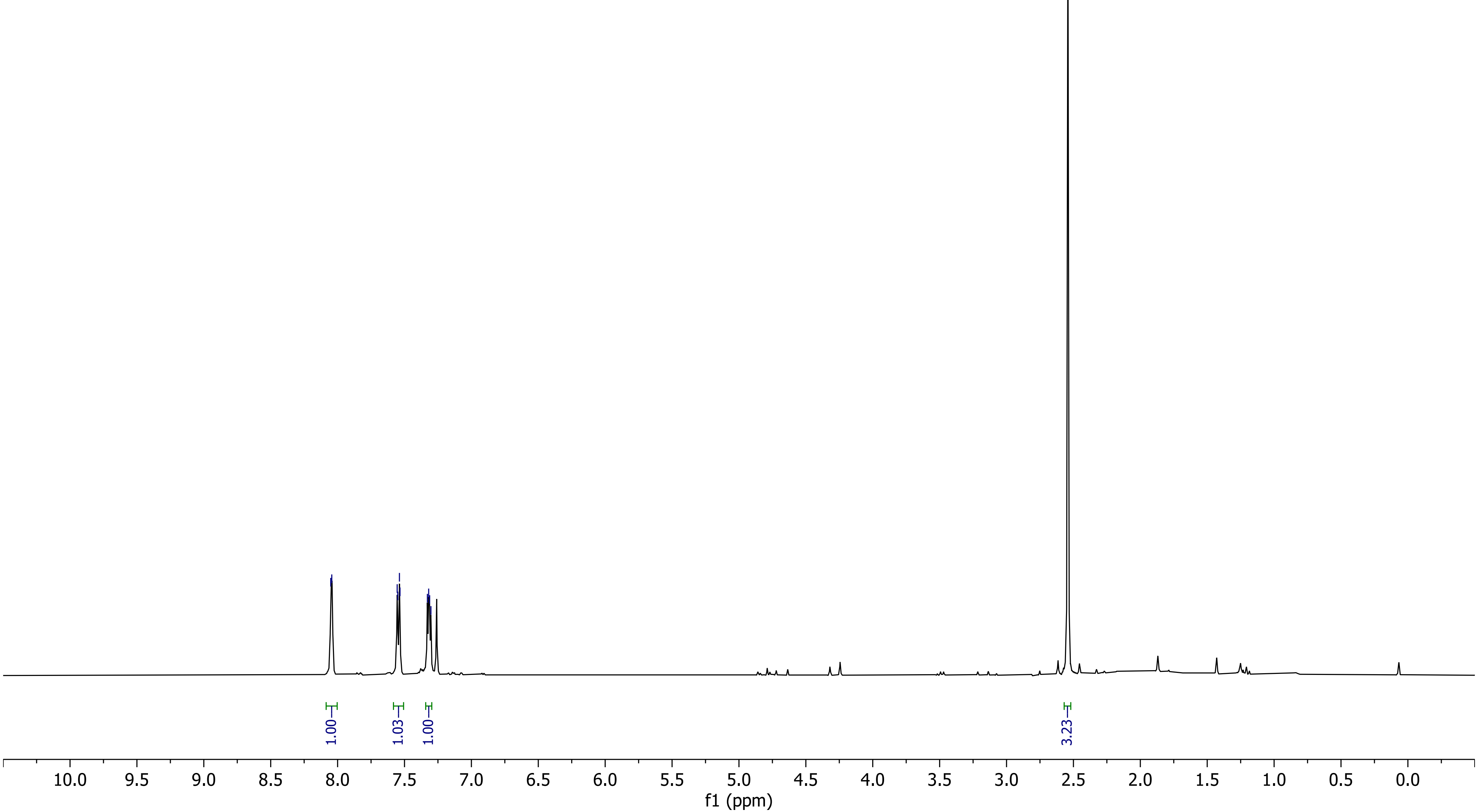

^1H NMR (300 MHz, $CDCl_3$)

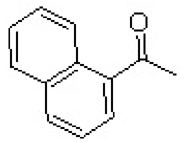

2o

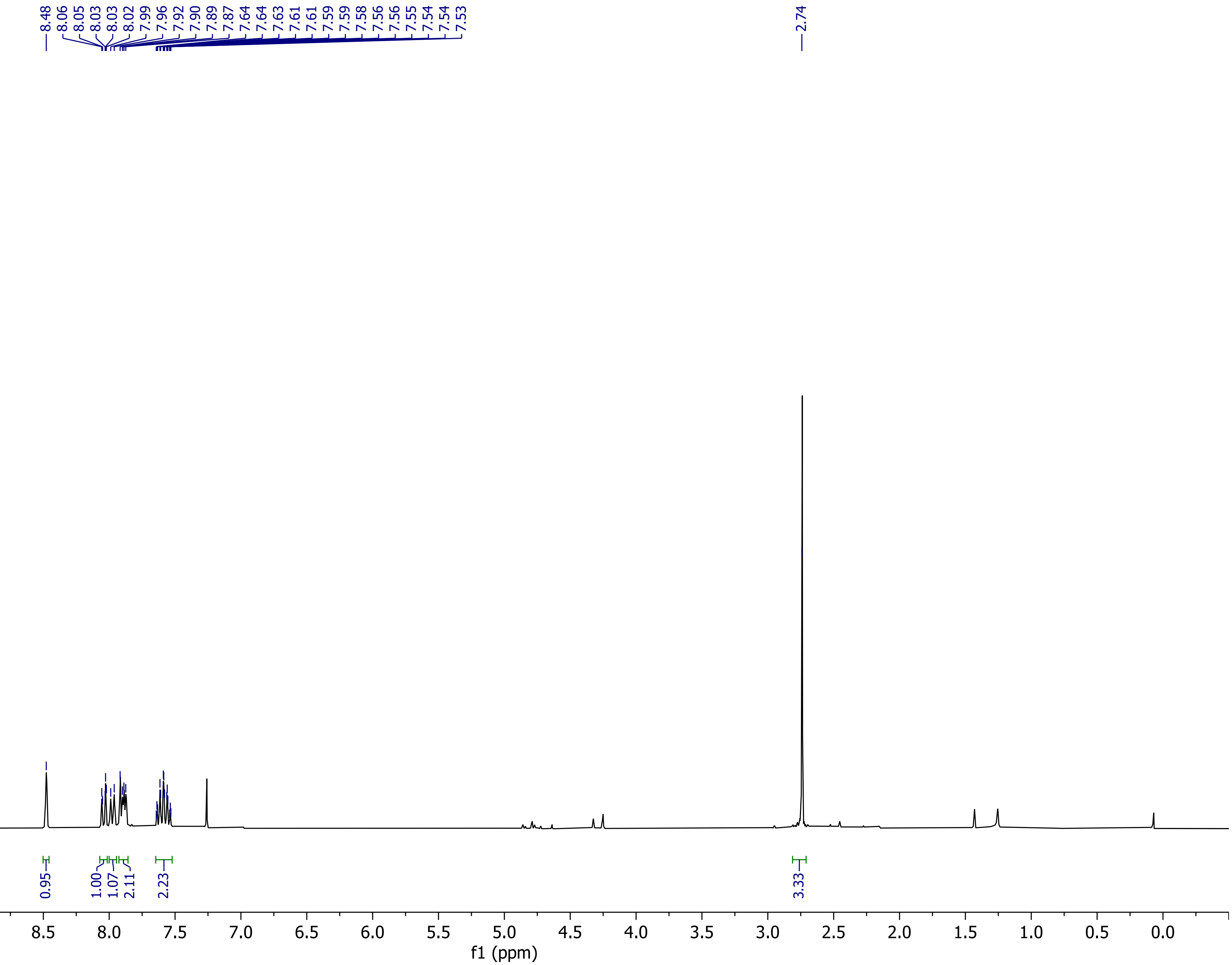

^1H NMR (300 MHz, $CDCl_3$)

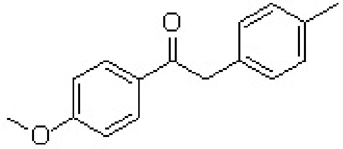

4a

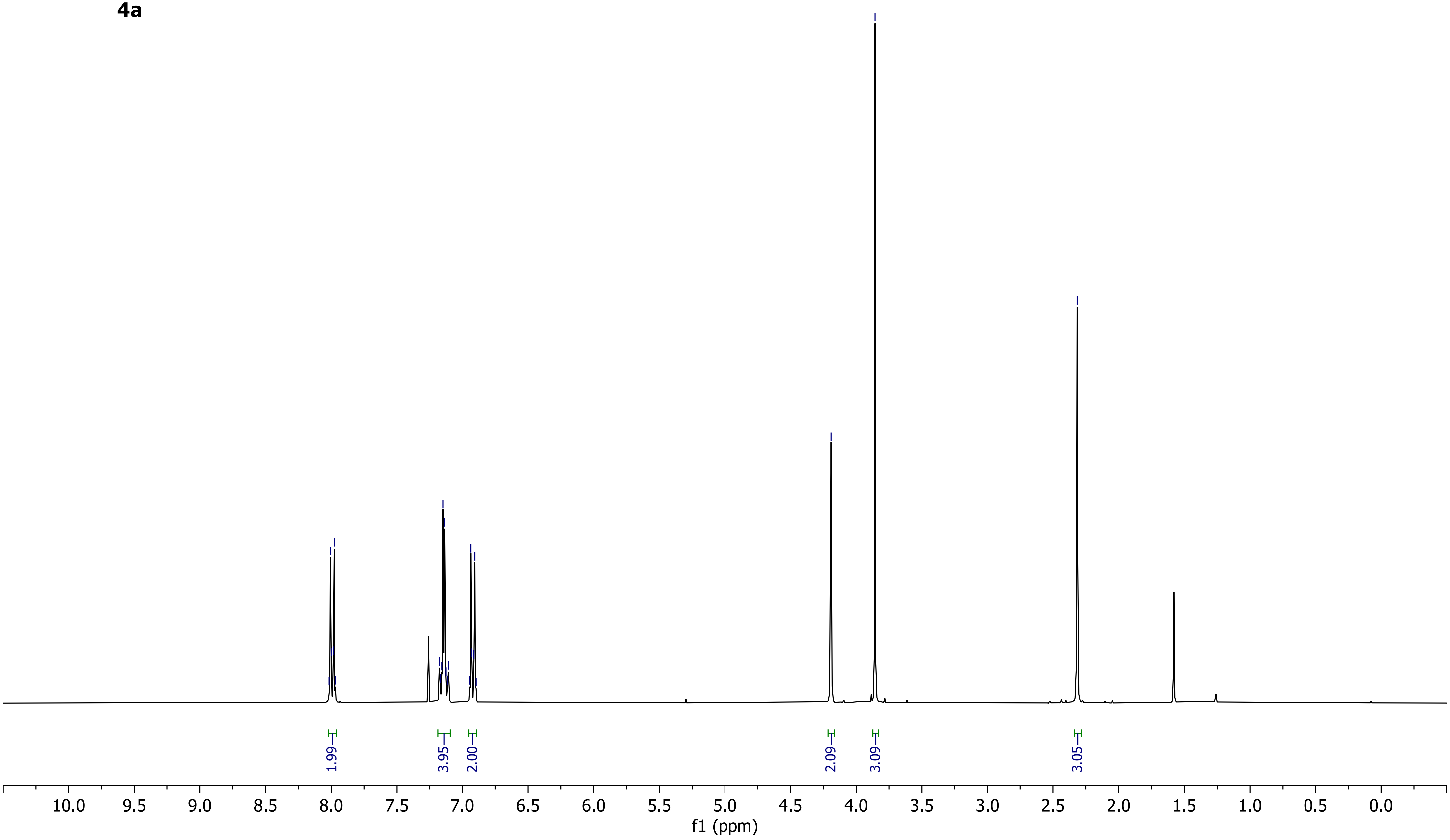

^{1}H NMR (300 MHz, $CDCl_3$)

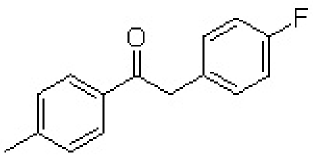

4b

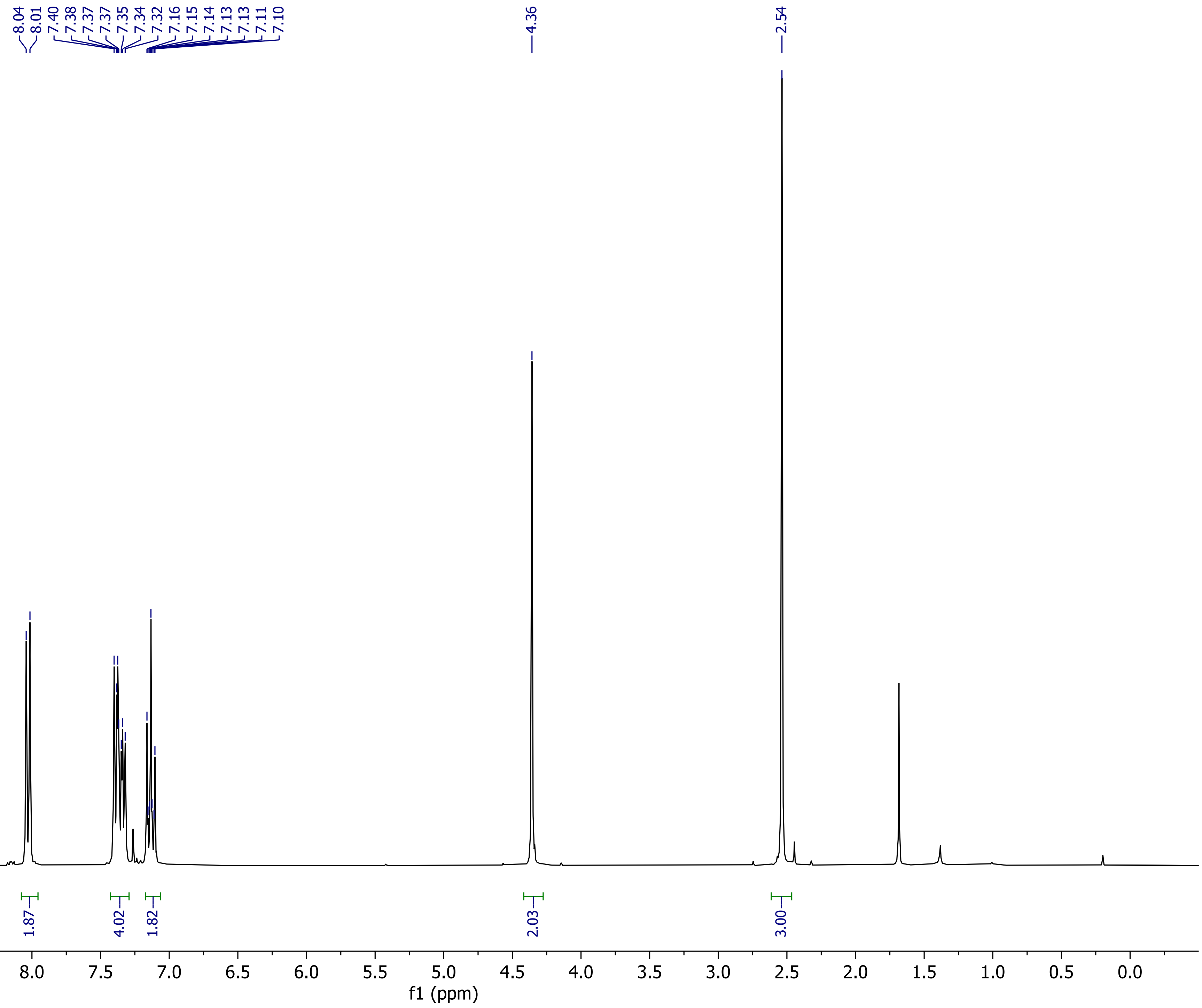

^{1}H NMR (300 MHz, $CDCl_3$)

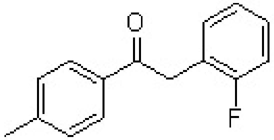

4c

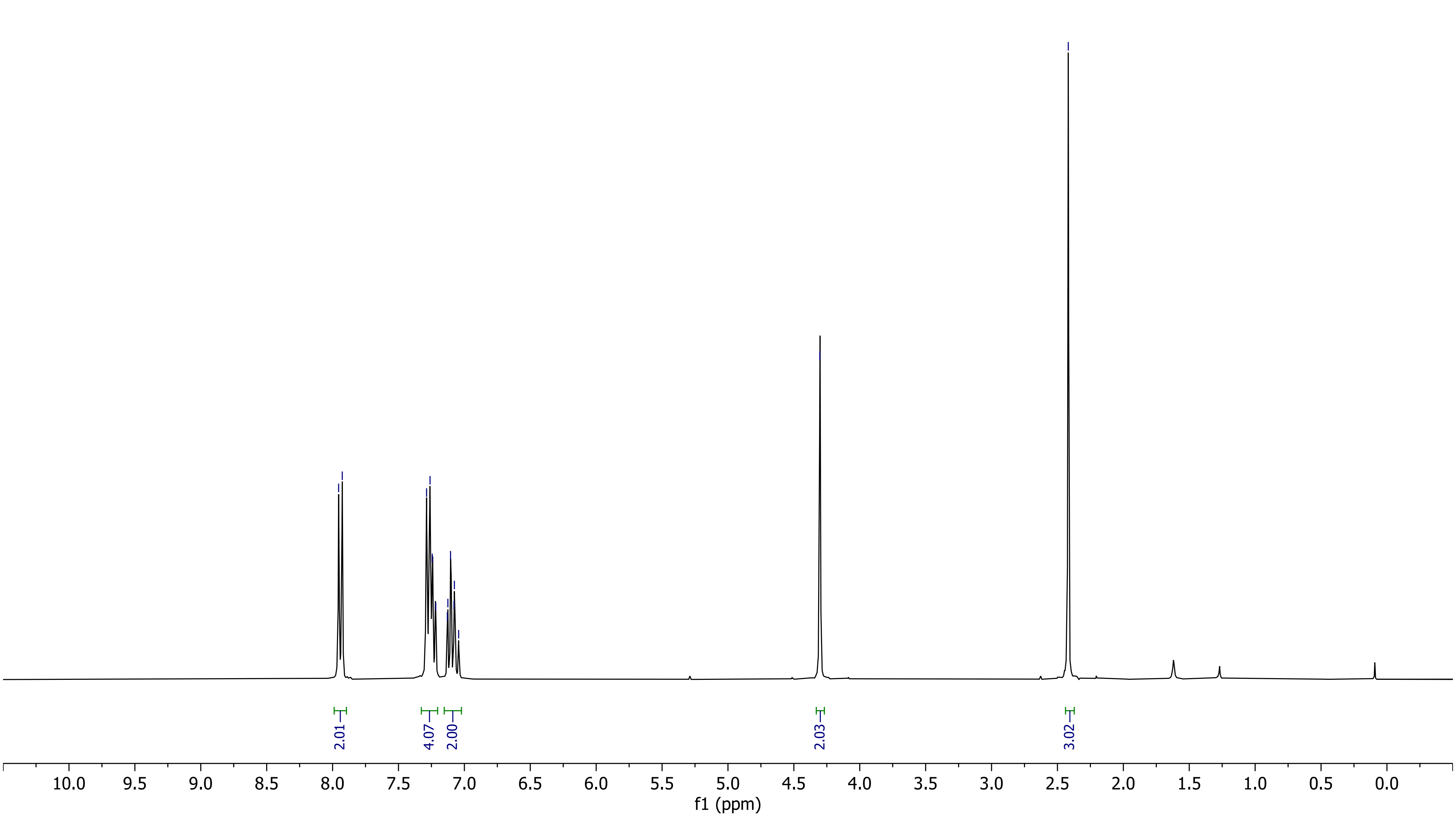

^{13}C NMR (75 MHz, $CDCl_3$)

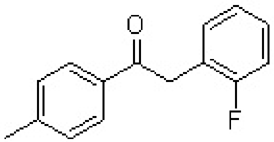

4c

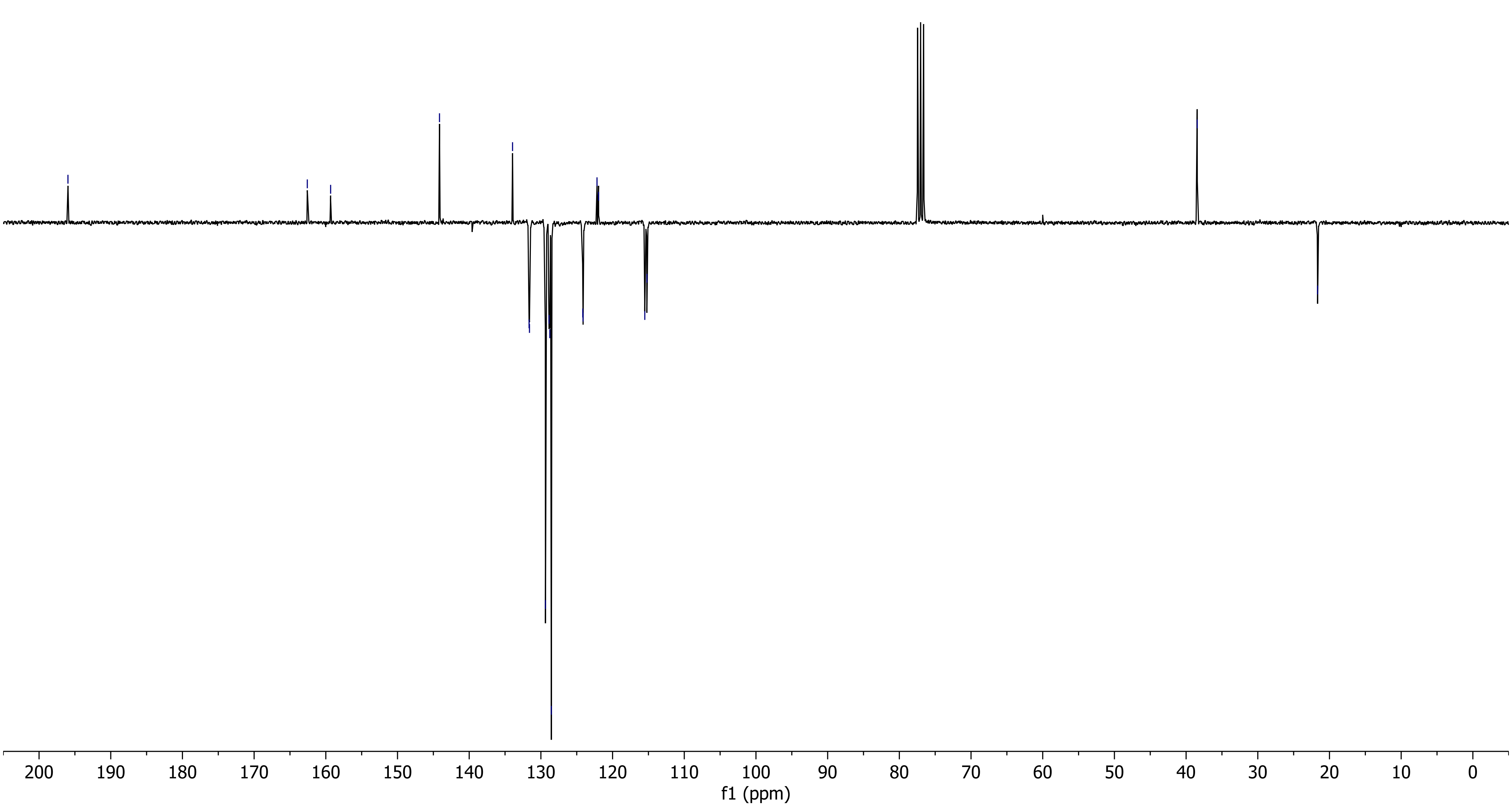

^{1}H NMR (300 MHz, $CDCl_3$)

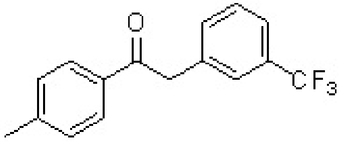

4d

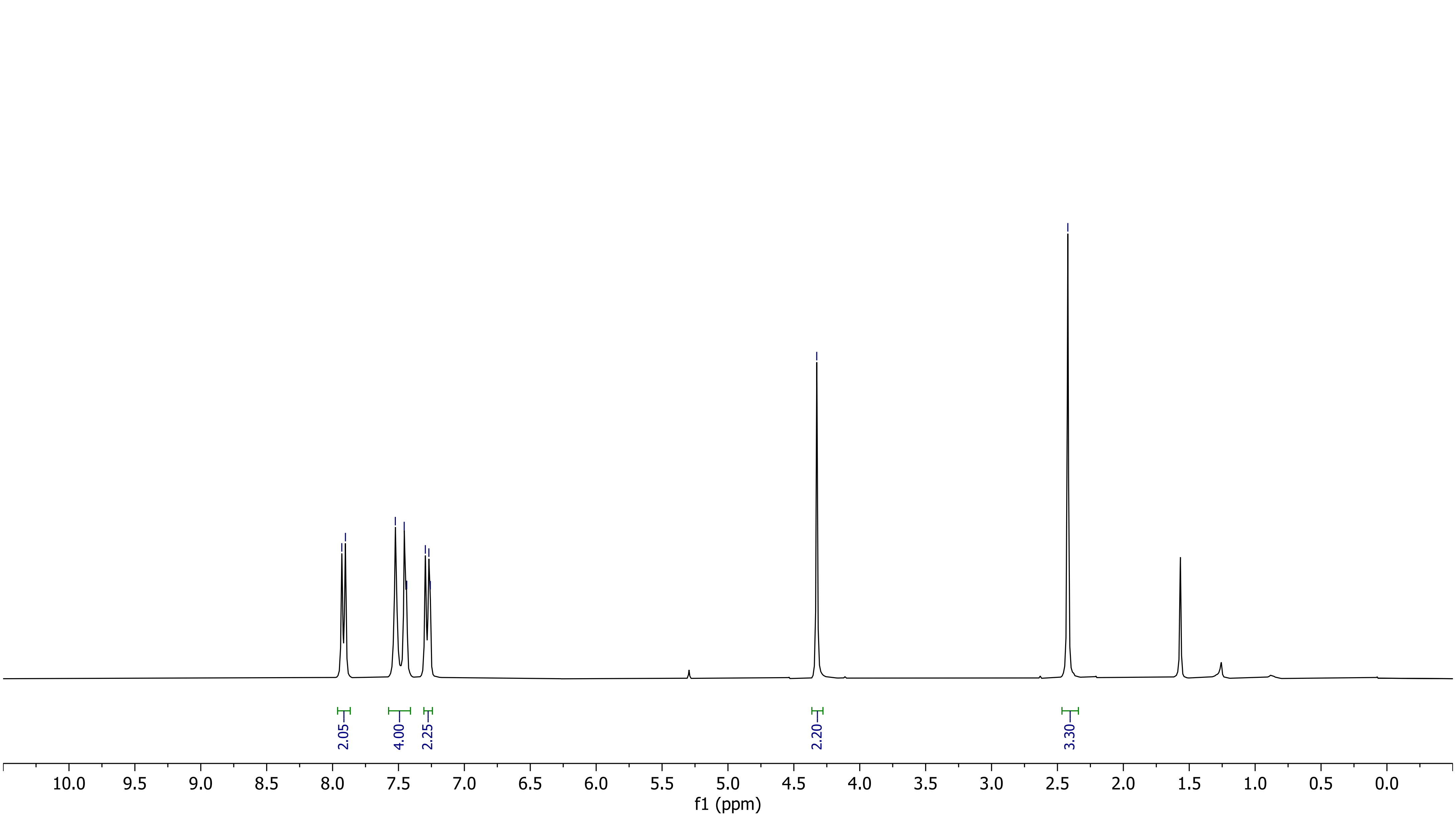

^{13}C NMR (75 MHz, $CDCl_3$)

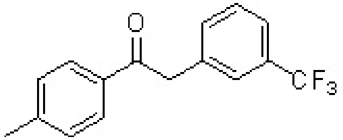

4d

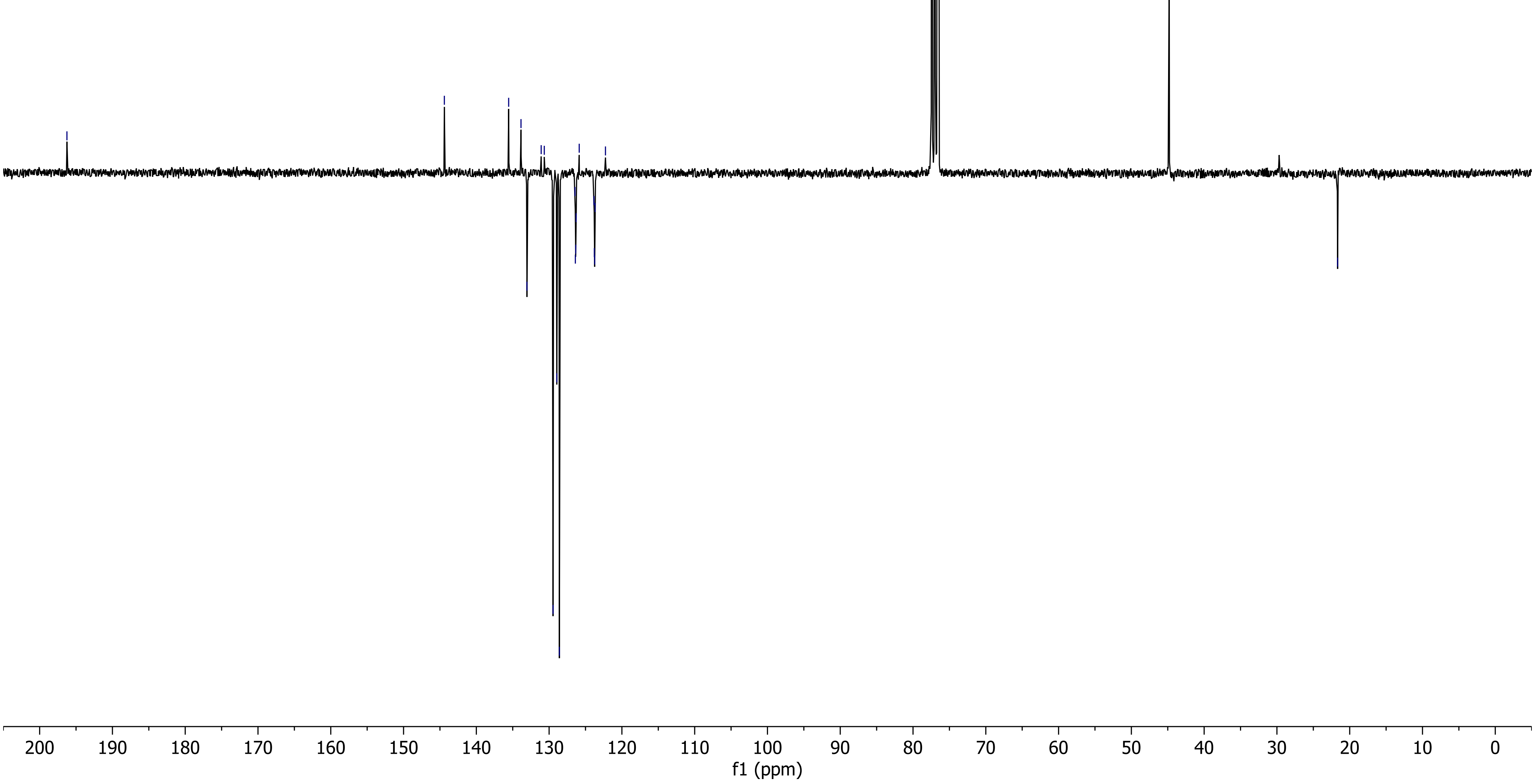

¹H NMR (300 MHz, $CDCl_3$)

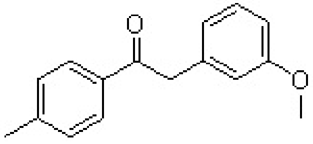

4e

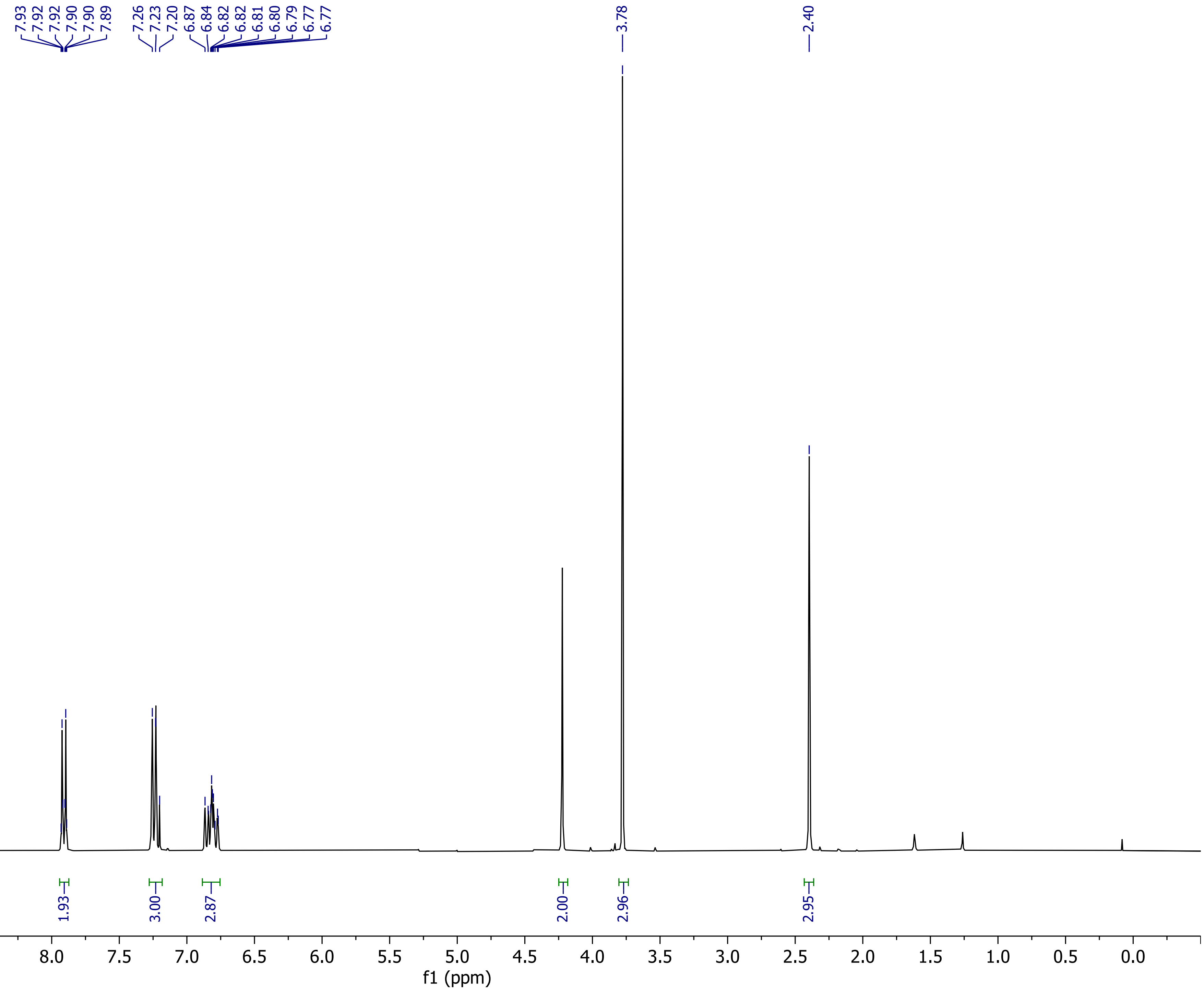

^{1}H NMR (300 MHz, $CDCl_3$)

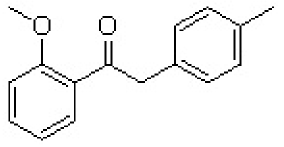

4f

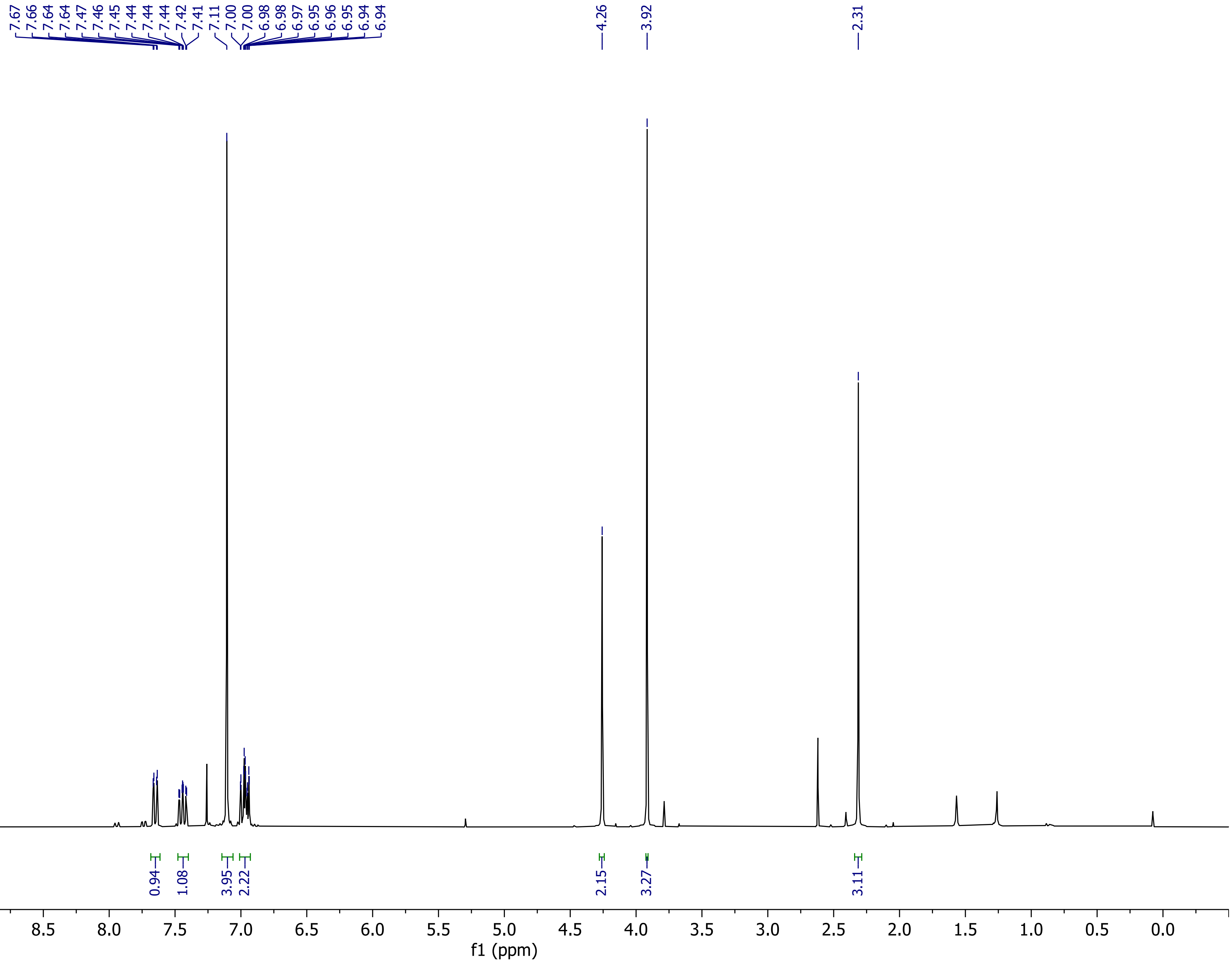

^{1}H NMR (300 MHz, $CDCl_3$)

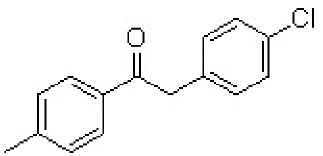

4g

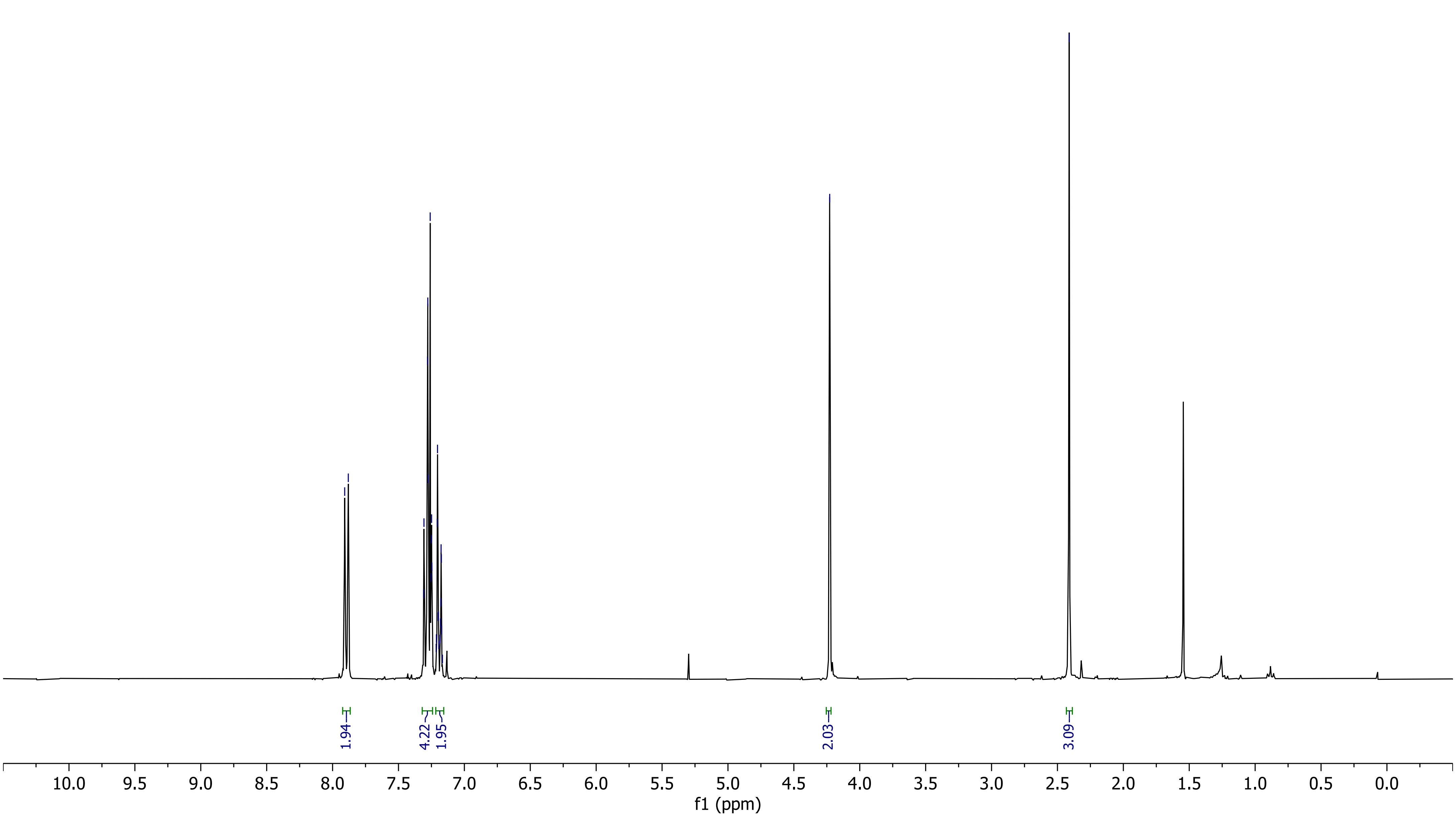

^{1}H NMR (300 MHz, $CDCl_3$)

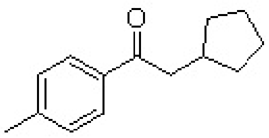

4h

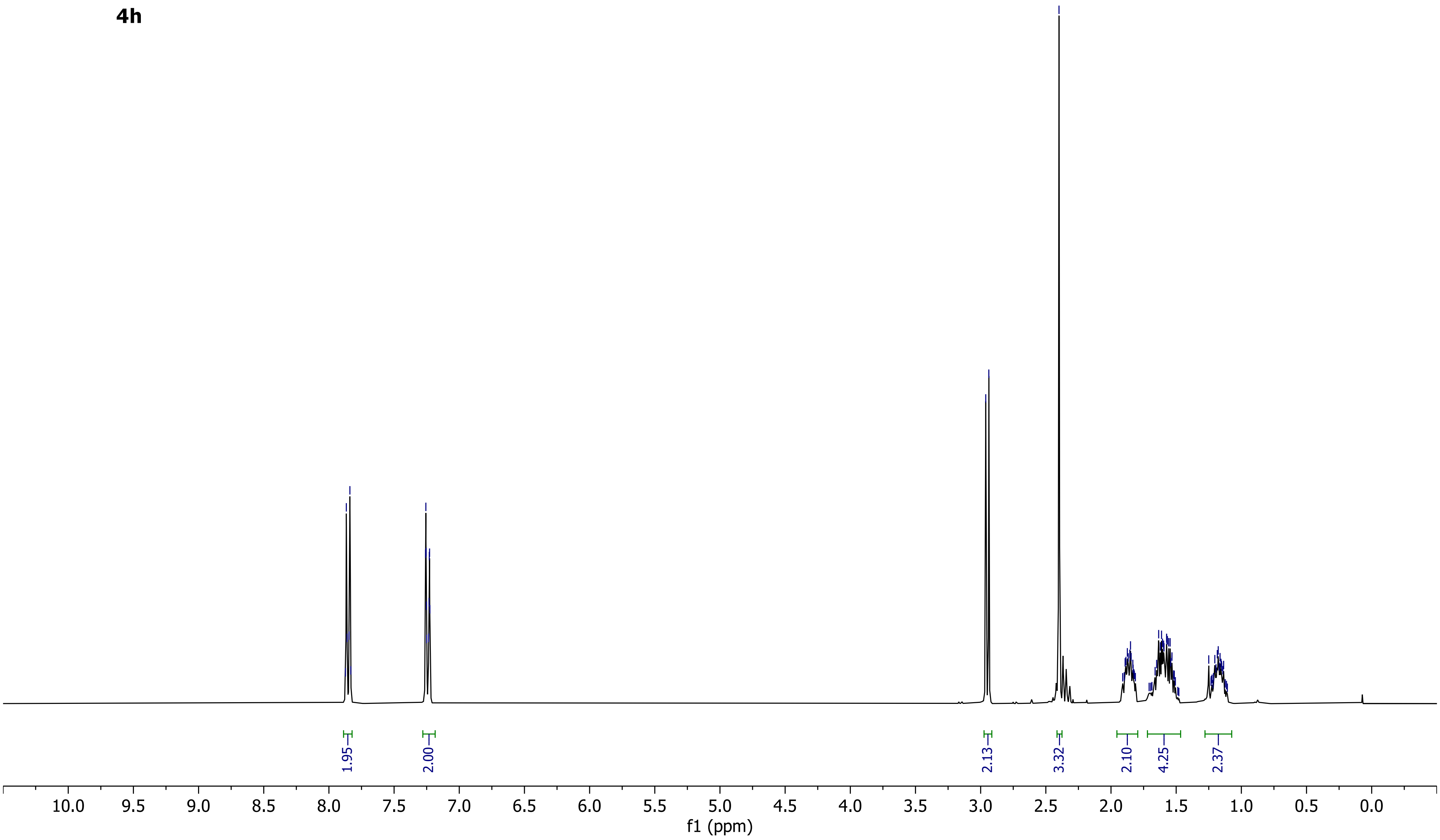

^{1}H NMR (400 MHz, $CDCl_3$)

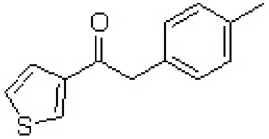

4i

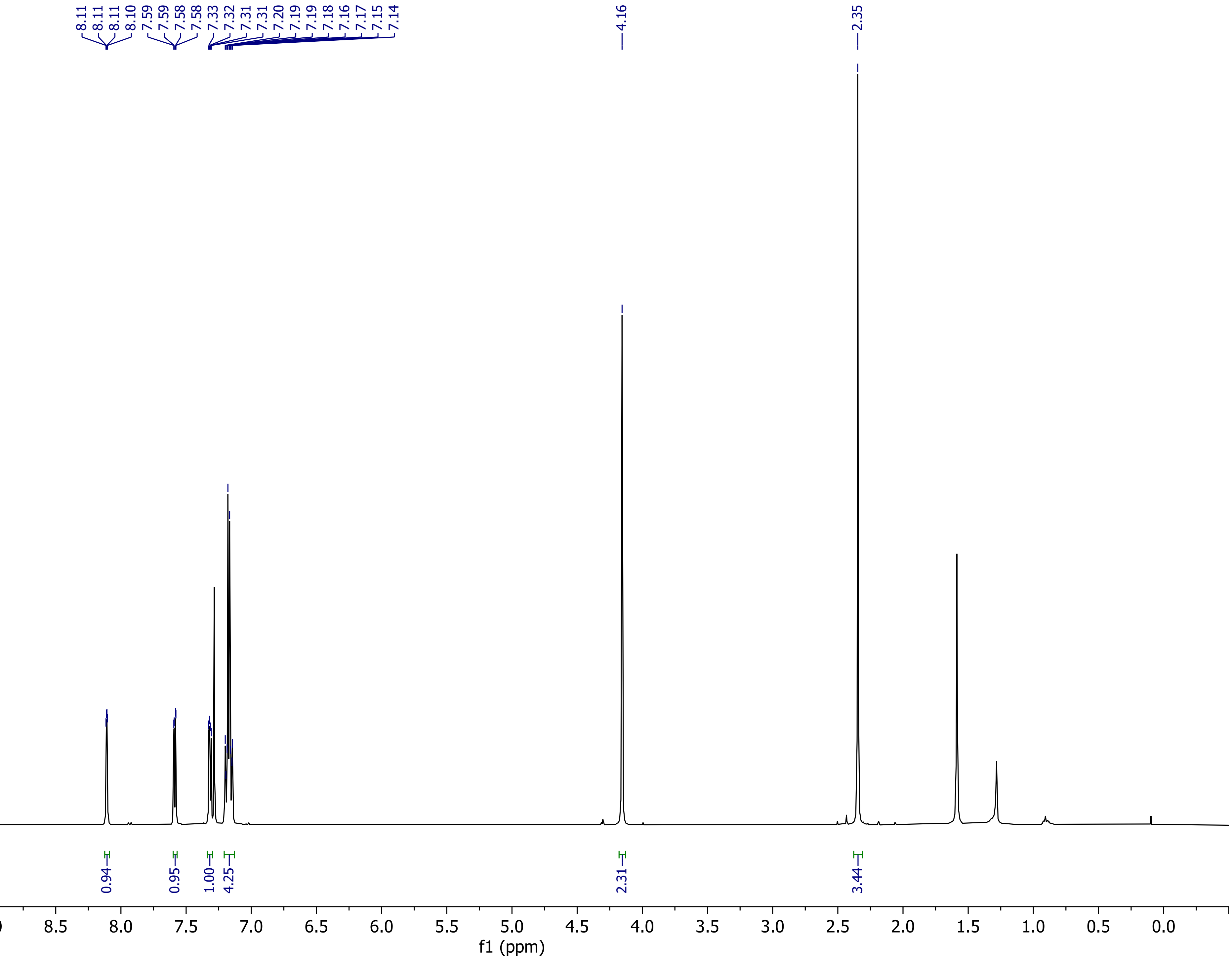

^{13}C NMR (101 MHz, $CDCl_3$)

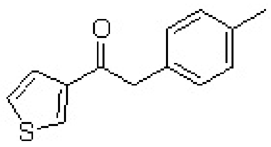

4i

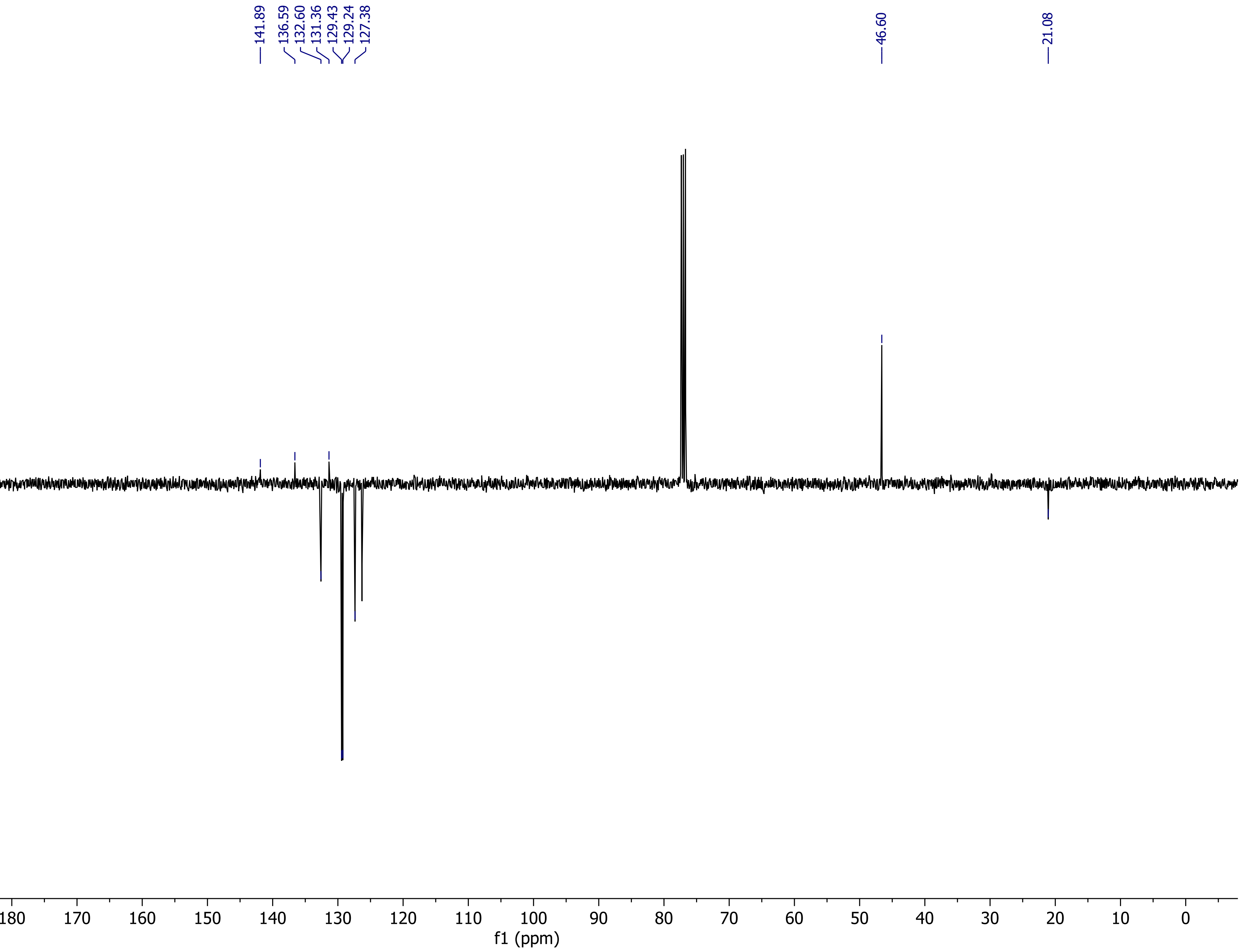

^{1}H NMR (300 MHz, $CDCl_3$)

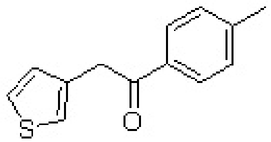

4i'

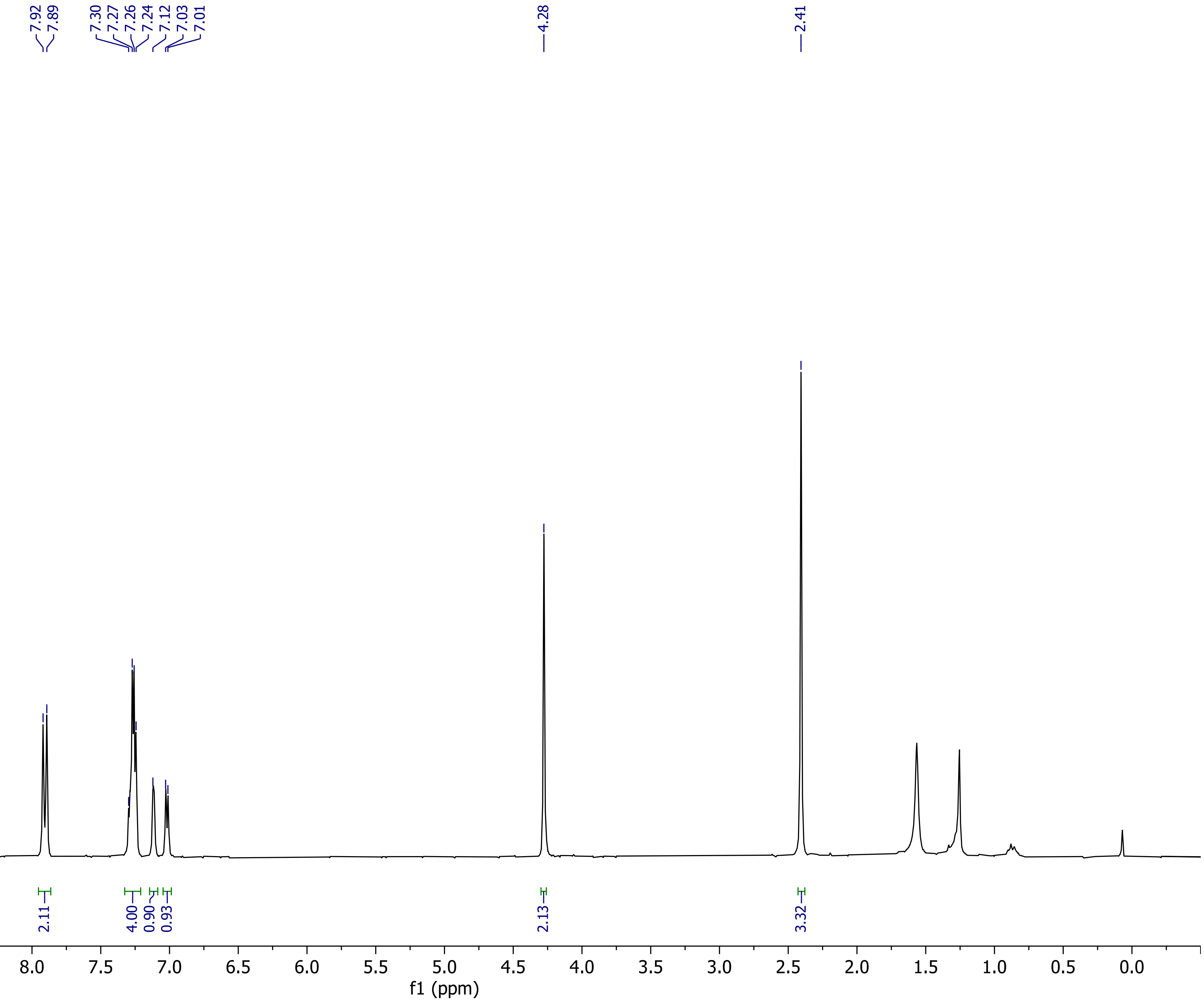

^{13}C NMR (75 MHz, $CDCl_3$)

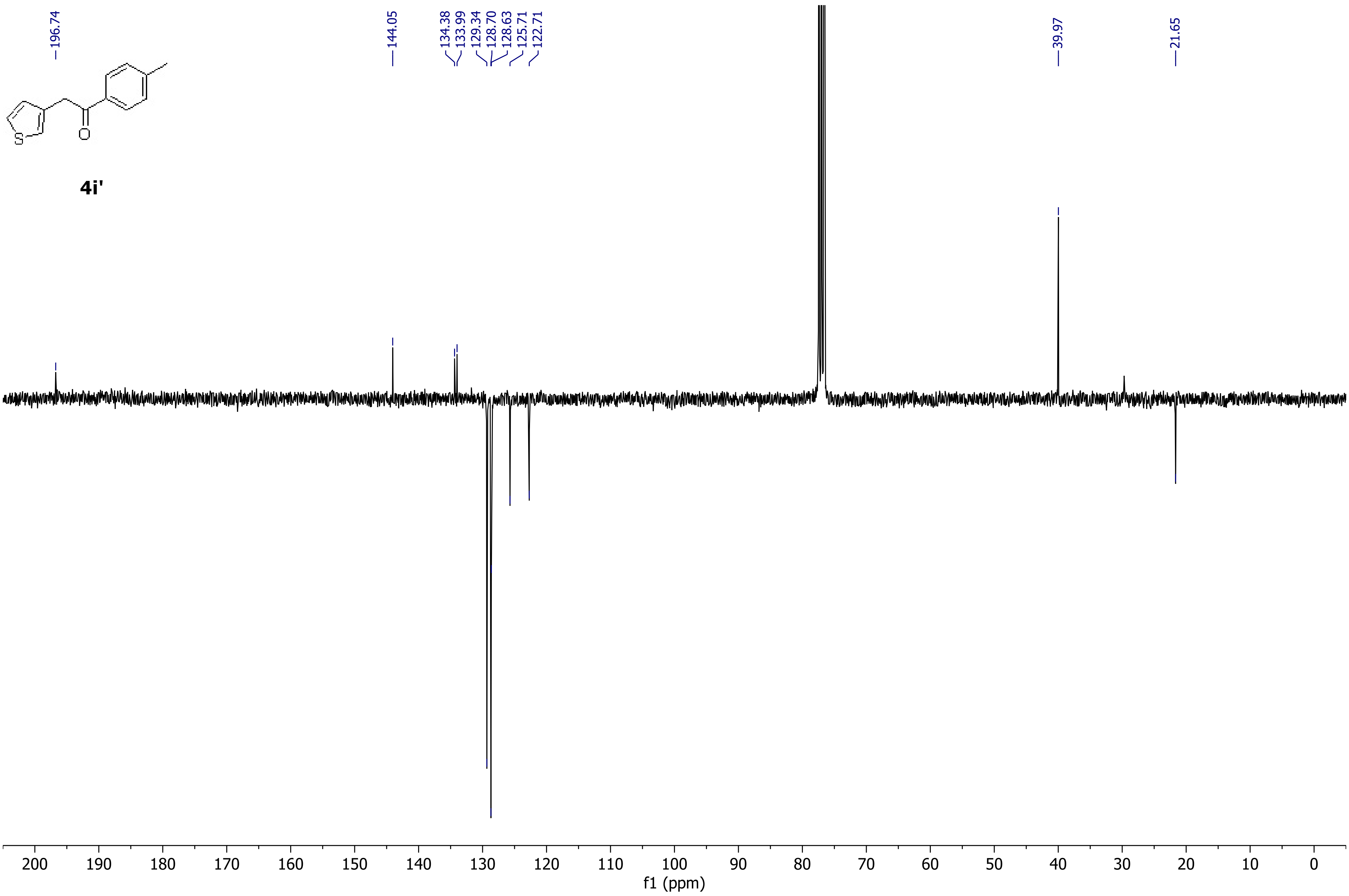

4i'
